# Hierarchical models of precedential constraint

**DOI:** 10.1007/s10506-025-09461-x

**Published:** 2026-03-09

**Authors:** Wijnand van Woerkom, Davide Grossi, Henry Prakken, Bart Verheij

**Affiliations:** 1 Department of Information and Computing Sciences, Utrecht University, Utrecht, The Netherlands; 2 Bernoulli Institute for Mathematics, Computer Science and Artificial Intelligence, University of Groningen, Groningen, The Netherlands; 3 Amsterdam Center for Law and Economics, Amsterdam, The Netherlands; 4 Institute for Logic, Language and Computation, Amsterdam, The Netherlands; 5 Present Address: Max Planck Institute for Comparative and International Private Law, Hamburg, Germany

**Keywords:** Case-based reasoning, A fortiori constraint, Explainable artificial intelligence

## Abstract

In recent years, models of *a fortiori* argumentation from the field of artificial intelligence and law, developed to describe legal case-based reasoning based on precedent, have been successfully applied to improve interpretability of data-driven decision systems. To aid with these applications, we further develop the theory of a fortiori case-based reasoning by extending the knowledge representations on which these models operate. More specifically, we modify the representations to accommodate incomplete information, as well as to incorporate both dimensional (as opposed to binary) and hierarchical (as opposed to unstructured) information. This results in four models—one for each combination of accommodating dimensional or hierarchical information. We investigate their formal properties, and find they are monotonic with respect to the addition of new precedents and of new facts, and that some are conservative extensions of other models. In addition, we exemplify each through a running example from the penitentiary law domain.

## Introduction

There has been a lot of interest over the past years in making artificial intelligence (ai) more interpretable or explainable, as this kind of technology is increasingly being applied to tasks with ethical, social, or legal impact to end-users. This emerging field is often called explainable artificial intelligence (xai), especially in the context of machine learning, where explainability is at issue.

It has been argued that one approach to tackle this problem is by considering the problems and solutions studied in the field of ai & law, of which explainability has always been a core aspect (Verheij 2020; Atkinson et al. 2020). Indeed, we have seen several applications of models of case-based reasoning from the ai & law literature to the improvement of interpretability of ai systems—in particular, the models of *a fortiori* reasoning put forth by Horty (2011, 2019). Starting with work by Prakken and Ratsma (2022), these models have been used as the basis of a post-hoc explanation method for black-box ai systems, and this work has since been continued by e.g. Peters et al. (2022, 2023); van Woerkom et al. (2022). Additionally, the model presented by Horty (2019) has been implemented as part of an interpretable decision support system at the Dutch national police force (Odekerken and Bex 2020). Furthermore, to aid with these applications, notions of *justifiability* and *relevance* were recently added to the theory by Odekerken et al. (2023). The present work is dedicated to advancing the theory of a fortiori case-based reasoning, so as to improve the capabilities of the aforementioned interpretability methods.

The model of a fortiori reasoning underlying these approaches, developed by Horty (2011), is called the *result model* (rm) of precedential constraint. It describes the type of reasoning performed by a court when it tries to adhere to past decisions called precedent cases. The model describes when a new decision is, or is not, consistent with respect to the precedents. In other words, it normatively describes the way in which a set of precedents constrains future decision-making. The rm works on the basis of a knowledge representation using *factors*—legally relevant fact patterns that are assumed to favor either a decision for the plaintiff or the defendant of the case.

Since its conception, two notable shortcomings of the rm have been pointed out. The first is that the factors used in the rm are binary, while in practice there may be relevant legal information which is encoded as a variable with more than two possible values, such as a natural or real number. To account for this, Horty (2019) presented an extended version of the rm which uses a knowledge representation that allows the legally relevant facts to take values in ordered sets called *dimensions*. For the purpose of comparison we will refer to this modified version of the rm as the *dimensional result model* (drm).

A second shortcoming of the rm, as already pointed out by Horty (2011), is that in practice factors often have a hierarchical structure, which the rm does not take into account. A court uses this hierarchical structure to move from low-level factors through a series of intermediate concepts, called *abstract* factors, before arriving at some final conclusion. This brings us to the first contribution of this work—to develop a *hierarchical result model* (hrm) that builds on the rm but operates on the basis of a *factor hierarchy* rather than a plain set of factors. The hrm should *subsume* the rm in the sense that any instance of the rm can be seen as an instance of the hrm with a trivial hierarchical structure.

**Fig. 1:**
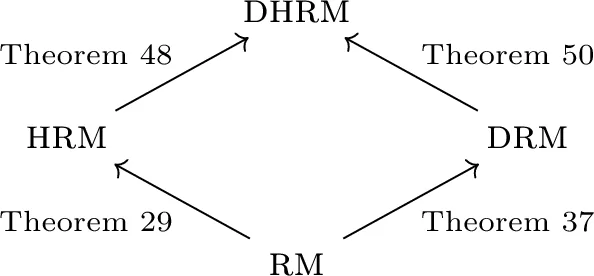
A graphical representation of the conservative extension relations between the models we introduce; e.g., the arrow from rm to drm indicates that all reasoning done in an instance of the rm can be faithfully reproduced in an instance of the drm

The rm and the drm have met with criticism with regards to their applicability when it comes to modeling the common law doctrine of precedent—see for example the work by Rigoni (2018), and Horty (2011) himself prefers the *reason model* over the rm when it comes to this task. However, the rm and extensions thereof can still be usefully applied more generally as a model of a fortiori reasoning—particularly in the context of explainable ai. The method of Prakken and Ratsma (2022), for example, uses the drm to generate an explanation in the form of an argumentative dialogue for a specific decision made by an ai system. This is done through an interpretation of *training examples as precedent cases*—the features of the training data are interpreted as dimensions in the sense of the drm, and the ai’s decision is subsequently checked against the training set according to the theory of the drm. The results of this check then form the starting point of an explanatory dialogue generated using argumentation theory. It is necessary to use the drm—as opposed to the rm—for this purpose, because machine learning data often uses non-binary features, and so are more naturally interpreted as dimensions. Therefore, in order for our proposed hrm to find application in xai, it should be expanded to operate on dimensions rather than factors. This is the second contribution of this work—to develop a dimensional hierarchical result model (dhrm) which operates on the basis of a *dimension hierarchy*. Ideally, the dhrm should subsume both the hrm and the drm, and in fact we are able to show this is formally true in our approach. Our contribution is summarized graphically in Fig. 1.

Following van Woerkom et al. (2022), we motivate and illustrate the applicability of the various a fortiori models discussed in this work by use of a running example from the legal domain of criminal sentencing. More specifically, we consider the tasks of judging recidivism risk and granting bail. We show that a fortiori reasoning is applicable to these tasks, and requires the use of a knowledge representation incorporating both dimensional and hierarchical information. Criminal sentencing is a highly relevant domain for our purposes—decisions surrounding criminal sentences have the potential to greatly affect peoples’ lives, and ai is increasingly being used to compliment or even replace human decision-making for these tasks.^1^

The contents of this work are structured as follows. In Sect. 2 we give an overview of related work. We then consecutively describe and analyze the rm, hrm, drm, and dhrm in Sects. 3, 4, 5, and 6. Having presented the models, we end in Sect. 7 with a conclusion and some ideas for future work.

## Related work

Much work has been done in the field of ai & law on the subject of case-based reasoning (cbr). Of particular relevance to the present work was the development of the hypo program, designed to generate legal arguments for justifying conclusions about who should win a dispute on trade secret law (Rissland and Ashley 1987; Ashley 1991). It does so chiefly on the basis of citations of prior cases called precedents. These cases are represented through the use of *factors*; in the words of Ashley (1991, Section 2):

Factors [...] are generalizations. Unlike rules, they do not specify necessary and sufficient conditions for a conclusion. Instead, they designate collections of facts, commonly observed in cases, that tend to strengthen or weaken a plaintiff’s argument in favor of a conclusion, such as a legal conclusion that the plaintiff has a trade secret.

It is difficult to define exactly what a trade secret is, and so factors are used to describe aspects of the situation at hand which make the case stronger or weaker for the party claiming to have the trade secret. A few examples of factors in this domain are^2^

- The extent to which the information is known to outsiders.
- The value of the information to the owner of the information, and to competitors.
- The amount of effort or money expended by the owner on developing the information.

The hypo program can generate a 3-ply legal argument for both sides of a dispute, by sequentially *analogizing*, *distinguishing*, and *rebutting* citations of precedent cases, on the basis of similarities and differences in the factor representations of the precedent cases and some novel problem situation. This innovative use of factors as a knowledge representation device, on the basis of which precedents can be compared and contrasted with novel fact situations, has become an essential aspect of many subsequent works.

Several programs akin to hypo were developed in its wake. What could be considered its immediate successor was the cato program—an intelligent learning environment designed to teach case-based legal reasoning to law students (Aleven and Ashley 1997; Aleven 2003). In many ways cato is comparable to hypo: it builds arguments for either side of a legal dispute by citing precedent cases, which are represented using factors. However, in contrast to hypo, the cato program constructs multi-case arguments organized around *issues*—the key legal questions that arise from the facts of a case, and that courts often address when explaining their decisions. It constructs such arguments through the use of middle-level normative background knowledge for the domain of law under consideration, which is represented as a *factor hierarchy*. The idea of such a hierarchy is that the factors that are used to represent cases provide support or opposition to higher level normative concepts called *abstract factors*. For example, the factor that the information under consideration in a trade secrets dispute is not known outside of the plaintiff’s business has influence on the more abstract factor that this information is valuable, which in turn bears on the legal issue of whether the information is a trade secret. Legal reasoning, according to this model, proceeds in a stepwise fashion from the lower level factors through the higher level factors, ultimately deciding on the legal issues, which then form the basis of a decision for either the plaintiff or the defendant. As hypo pioneered the use of factors as a knowledge representation device for cases, so did cato pioneer the use of factor hierarchies. Continuing this line of work, the ibp and vjap programs were presented by Bruninghaus and Ashley (2003); Grabmair (2017), developed for the purpose of predicting legal case outcomes, and both of which operate on the basis of factor hierarchies.

Aside from spurring the further development of programs designed to produce legal arguments and/or predict case outcomes, hypo has also inspired work of a more theoretical character, and in particular to some works focused on giving an account of *precedential constraint*—the way in which precedent cases constrain future decision-making according to the *stare decisis* principle. Notably, building on the reason-based logic of Hage and Verheij (1994) and the formal theory of legal cbr by Prakken and Sartor (1998); Roth (2003) represented cases using a logical language and formulated a fundamental *a fortiori* principle of constraint. This principle roughly states that in a novel fact situation the same decision should be reached as in a precedent case, if the novel fact situation has equal-or-more support for that decision than the precedent case did, as measured by their factor representations. Concurrently, Horty (2004, 2011) concisely modeled this same principle through his *result model* (rm) of precedential constraint, using a ‘plain’ factor-based representation of cases. The rm, and extensions thereof, have been influential in the literature on formal precedential constraint, and are the main focus of the present work.

As noted above in the introduction, a downside of the factor-based approach to case representation is that not all legally relevant information can be captured as a binary proposition—in many instances the legally relevant information is multivalued. As a matter of fact, hypo did allow for the representation of multivalued information in the form of what its authors called *dimensions*, but many of the subsequent works that sprung from hypo, such as cato, only used factors. Consider as an example of a dimension in the trade secrets domain the question of whether the alleged secret has been disclosed to people outside the plaintiff’s business. One way to represent this is as a binary proposition, i.e. a factor, stating whether the secret has been disclosed or not. However, a more fine-grained representation would allow the exact number of disclosees to be specified—i.e. as a dimension. Dimensions, like factors, are assumed to be legally relevant in the sense that the possible values in a dimension have an influence on who should win a dispute; but, unlike factors, it may be more difficult—or even impossible—to say which side is favored by a specific value.

This led Horty (2019) to adapt his result model of precedential constraint to operate on the basis of a case representation using dimensions as opposed to factors. For the sake of discussion we will call this version the *dimensional result model* (drm). His idea for modelling the preference of values in a dimension towards an outcome was to use a (partial) ordering: a value in the dimension is ‘greater than’ another if it provides greater (defeasible) support for the plaintiff. In other words, in Horty’s model, values do not directly favor an outcome, but instead may be more or less in favor of an outcome compared to other values. For example, in the aforementioned case of the number of disclosees in a trade secrets dispute, the smaller the number of disclosees, the stronger the case becomes for the plaintiff; and conversely, the more disclosees, the better it is for the defendant. Cases in the drm are modelled as assignments of values to all the dimensions describing the legal domain under consideration, and precedential constraint is defined using the same a fortiori principle that is used for the plain rm: an outcome is forced for a side by a precedent case if the novel fact situation has equal-or-greater support for that side.

The drm was conceived primarily as a formal model of legal cbr, though, as mentioned, it has been criticized in regards with its capacity to do this, and alternatives have since been proposed by e.g. Rigoni (2018), among others. However, the drm has also found applications in explainable artificial intelligence (xai), based on the analogy of *training examples as cases*: just as a court draws on precedent cases to decide on a novel fact situation, machine learning systems draw on training data to decide on novel data points. Based on this analogy, Prakken and Ratsma (2022) combined the drm with hypo style argumentation to develop a method for explaining the decisions of data-driven machine learning systems. Peters et al. (2022, 2023) then further improved this method, by making it more robust to inconsistent data, and less reliant on the availability of the training data of the ai system. The drm has also been implemented as part of a human-in-the-loop decision support system for classification of fraudulent web shops at the Dutch national police lab ai (Odekerken and Bex 2020; Odekerken et al. 2023). To facilitate this and similar applications, Odekerken et al. (2023) have further developed the theory of the drm by adding formal notions of *justification*, *stability*, and *relevance*. These notions allow the model to be applied to fact situations in which the values of the dimensions are not known precisely, but are only known to lie within a certain subset. Lastly, we mention that case-based reasoning techniques are also used more broardly in the ai & law literature in conjunction with argumentation theory to the development of xai methods, such as e.g. by Čyras et al. (2016, 2019).

In sum, models of cbr that stem from the literature on ai & law are increasingly being used for the development of xai methods and systems, and these applications are being complemented by further theoretical research. This is also the goal of the present work: to advance the theory of legal cbr, so that xai methods and systems based on this theory become more expressive. We do so by first extending Horty’s rm, which uses a plain factor-based representation of cases in the style of hypo, to make use of a hierarchical factor-based representation in the style of cato; we call the resulting model the *hierarchical result model* (hrm). Then, just as Horty extended his rm to the drm in order to account for dimensional information, we extend the hrm to account for dimensional information; we call the resulting model the *dimensional hierarchical result model* (dhrm).

We conclude by mentioning that variations of the *reason model* of precedential constraint (in contrast with the rm), introduced by Horty (2011), have also been explored in the context of hierarchical constraint (Bench-Capon and Atkinson 2021; Bench-Capon 2023; Canavotto and Horty 2023a, b; Bench-Capon 2024). The reason model expands on the result model by encoding an additional reason for the decision by the court, in the form of a subset of the factors favoring the side for which the court decided. We opt to focus on the result model, rather than the reason model, because in our understanding these reasons do not have an evident place in the interpretation of training examples as cases. Whereas court documents might include a description of such a reason, machine learning training data typically consists of plain feature data together with a label, without any description of which features were, or were not, relevant for the label associated to the training example. This makes it more difficult to apply the reason model to the development of xai methods, but it has seen applications in this field—Canavotto and Horty (2022) apply the reason model in the context of computational normative reasoning, and Di Florio et al. (2023) apply it towards the development of counterfactual explanations of ai decisions. Since machine learning data are often non-binary, it is natural to use a dimension-based version of the reason model for such applications. Horty (2019) has proposed such an extesion, but ultimately this version proved to be equivalent to the drm. Alternatives have been proposed to rectify this, e.g. by Rigoni (2018), who treats dimensions as ordered sets of reasons. Applications of the reason model to xai should look to these dimension-based extensions, but as mentioned, we base the present work on the result model, as opposed to the reason model.

Lastly, we note that there are other hierarchical models of constraint in the literature, pertaining to the hierarchical structure that is present in most judicial systems (Broughton 2019; Di Florio et al. 2024). In the present work, we use the term *hierarchical* to refer to the hierarchical structure of factors and dimensions.

## The result model

We start by discussing Horty’s (2004; 2011) result model of precedential constraint. In Sect. 3.1 we explain the concept of factors through the use of a running example based on the legal domain of criminal sentencing. We then formalize the knowledge representation used by Horty’s model in Sect. 3.2, and subsequently give his notion of constraint in Sect. 3.3. In Sect. 3.4, we generalize Horty’s theory by allowing fact situations to assign a truth value to only a subset of the factors of the given domain, and adapt the notion of constraint to this setting. We refer to the resulting model as the rm, and it will serve as the basis for the models we present in the subsequent sections. We then conclude our discussion on the rm in Sect. 3.5 by considering whether the type of reasoning represented by the rm is *monotonic* or not—a property which characterizes the defeasibility of inferences, and plays an important role in the study of reasoning and argumentation (Strasser and Antonelli 2019). We consider two types of monotonicity; that in the addition of new cases to the case base, and that in the addition of new information to the focus fact situation. We find that the rm is monotonic with respect to both of these types of additions.

### An example of factors

We illustrate the various kinds of models discussed in this work through a running example on the criminal sentencing domain. In this case, we consider a judgement of whether a convict is at low or high risk of recidivism—a primary task of the compas software. Much research has been done on the factors influencing recidivism; see e.g. the work by Yukhnenko et al. (2020) for a recent meta-study. Below is a graphical representation of a number of such factors:

**Figure:**
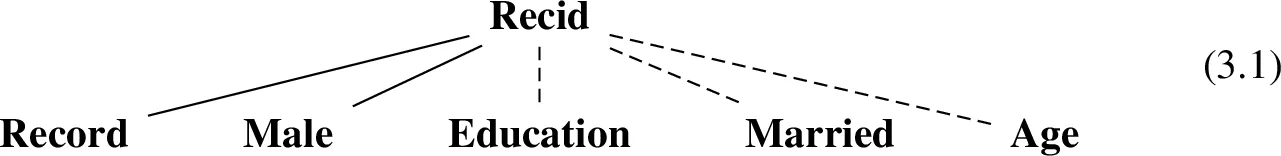

The factors in the bottom row respectively indicate whether the defendant has a criminal record, is male, has a high school diploma, is married, and is over the age of 21. A solid line between a factor and the ${\textbf {Recid}}$ node indicates that the presence of that factor suggests a higher risk of recidivism, while a dotted line indicates that its presence suggests a lower risk. For instance, having a criminal record indicates a higher risk, while being married indicates a lower risk. Now, suppose a 30 year-old unmarried male defendant with a pre-existing criminal record and no high school diploma was judged to be at high risk of recidivism. Given our assumption that older people tend to recidivate less, it follows *a fortiori* that a defendant who is on all accounts similar, but is 20 years old instead of 30, should also be judged to be at high risk of recidivism.

### Knowledge representation

A *factor* is a propositional variable, i.e. a variable which is either true (denoted $\textbf{t}$) or false (denoted $\textbf{f}$). We denote factors using lowercase letters *p*, *q*, *r*, etc. The domain is modeled by a *factor partition* $F = {{\,\textrm{Pro}\,}}\cup {{\,\textrm{Con}\,}}$, so ${{\,\textrm{Pro}\,}}$ and ${{\,\textrm{Con}\,}}$ are sets of factors satisfying ${{\,\textrm{Pro}\,}}\cap {{\,\textrm{Con}\,}}= \emptyset$. A *fact situation* is a valuation of *F*, i.e. a function $X: F \rightarrow \{\textbf{t}, \textbf{f}\}$ assigning true or false to every factor in *F*. We use upper case letters *X*, *Y*, *Z* etc. to denote fact situations, and write X⊨p for $X(p) = \textbf{t}$ and $X \vDash \lnot p$ for $X(p) = \textbf{f}$. We may combine statements of this form with set notation, so for instance by $X \vDash \{p, \lnot q, r\}$ we mean X⊨p, $X \vDash \lnot q$, and X⊨r.

Cases are decided for either of two *sides*; the plaintiff, denoted by π; or the defendant, denoted by δ. Each factor p∈F has a *preference* for exactly one of the two sides, which is modeled by two sets ${{\,\textrm{Pro}\,}}$ and ${{\,\textrm{Con}\,}}$, which represent the factors supporting or opposing a decision for the plaintiff, respectively. If a factor is pro-π (δ) we assume it is con-δ (π). A *case* is a pair (*X*, *s*) with *X* a fact situation and *s* a side; a *case base* $\mathcal {C}$ is a finite set of cases.

### Constraint

The idea behind the rm is that a decision of a fact situation *X* for a side *s* constitutes a balancing of the pro-*s* factors in *X* against the con-*s* factors in *X*. The support that factors provide for an outcome is defeasible and unquantified, which makes it difficult to weigh sets of pros against sets of cons. However, once a set of pros was deemed to outweigh a set of cons, any superset of the set of pros should also outweigh any subset of the set of cons. This intuition is formalized by the following definition.

#### Definition 1

Let *X* be a fact situation and $\mathcal {C}$ a case base, then *the decision of X* *for* π *is forced by* $\mathcal {C}$, denoted $\mathcal {C}, X \vDash \pi$, if and only if there is a case $(Y, \pi ) \in \mathcal {C}$ such that:

- for all $p \in {{\,\textrm{Pro}\,}}$: if Y⊨p then X⊨p, and
- for all $p \in {{\,\textrm{Con}\,}}$: if X⊨p then Y⊨p.

Likewise, *the decision of* *X* *for* δ *is forced by* $\mathcal {C}$, denoted $\mathcal {C}, X \vDash \delta$, iff there is a case $(Y, \delta ) \in \mathcal {C}$ such that:

- for all $p \in {{\,\textrm{Con}\,}}$: if Y⊨p then X⊨p, and
- for all $p \in {{\,\textrm{Pro}\,}}$: if X⊨p then Y⊨p.

Note that the rm was not necessarily designed as a method for weighing pros and cons against each other. Instead, it normatively prescribes a principle of what it means for such a weighing method to act in accordance with the a fortiori principle with respect to some set of previous cases. For example, given the factors depicted in graph (3.1), the rm is not intended to classify a given defendant as low or high risk. Instead, it prescribes to such a decision-maker, such as in this example a court, or the compas software, what it means to act in accordance with the precedent and the a fortiori principle. This is also closely related to the work by Canavotto and Horty (2022), who present an approach to computational normative reasoning based on Horty’s reason model.

#### Example 2

We demonstrate Definition 1 of constraint through an example based on the factors in (3.1). Let *Y* be a fact situation specified by $Y \vDash \{{\textbf {Record}}, \lnot {\textbf {Male}}, \lnot {\textbf {Education}}, {\textbf {Married}}, \lnot {\textbf {Age}}\}$, which is part of a case (*Y*, 1), where 1 represents a judgement of high recidivism risk. In other words, the case (*Y*, 1) represents a decision that the person described as fact situation *Y* was deemed to pose a high recidivism risk by some decision-maker. When does this precedent constrain the risk assessment of a new fact situation *X*? Unfolding the definition we find:Indeed, when *X* matches the truth status of all the factors that were indicative of a recidivism risk in *Y*, then *X* should also be judged to be at a high risk. The truth status in *X* of the other factors is relevant—for example, with respect to the comparison with *Y* it does not matter whether *X* is married or not, because if *X* is not married then this will only further strengthen the case for a “high risk” assessment. However, if *X* fails to match the status of, say, ${\textbf {Record}}$, then it may be argued that *X* has less support for a “high risk assessment”—compared to *Y*—and thus the precedent case (*Y*, 1) should not constrain *X*.

### Partial fact situations

The account of the rm that we have given here differs slightly in form compared to how it was introduced by Horty (2011), who models a fact situation *X* as a subset X⊆F of the factors. Of course, this is formally equivalent to using a valuation (or, *indicator function*) $X: F \rightarrow \{\textbf{t}, \textbf{f}\}$; given a subset X⊆F we can define a valuation assigning $\textbf{t}$ to p∈F if p∈X and $\textbf{f}$ otherwise, and conversely, given a valuation $X: F \rightarrow \{\textbf{t}, \textbf{f}\}$ we can define a subset $\{p \in F \mid X(p) = \textbf{t}\} \subseteq F$.

These representations have a downside: for any particular fact situation we are forced to say, for each factor, whether it applies in the situation or not. This is not entirely realistic, because in practice it may be unknown what the truth value of a factor is. It may be argued that, for the subset representation, the meaning of the absence of a factor from the subset is that it is unknown whether it applies or not. However, this interpretation has the same problem of not being sufficiently fine-grained; if, for example, the **Married** factor is absent from a subset, does this represent that the defendant is unmarried, or that it is not known whether the defendant is married?

The need for a more fine-grained representation will become all the more pressing once we move from a plain set of factors to a factor hierarchy, and so we will consider a modification to the knowledge representation of the rm that accounts for unassigned factors. We do so by considering *partial* fact situations, in the sense of partial functions on sets. A partial function of a set *A* to a set *B* is a function f:C→B for some subset C⊆A. Given a partial function on a set *A* we write ${{\,\textrm{dom}\,}}(f) \subseteq A$ for the subset of *A* on which *f* is defined. Applying this definition to valuations yields the definition of a partial fact situation *X*: a valuation that is only defined on some subset G⊆F of factors. Note that, according to this definition, a situation *X* which is defined on all factors (so ${{\,\textrm{dom}\,}}(X) = F$) is also considered a partial fact situation. To differentiate these from strictly partial fact situations we will call such fact situations *complete*. We maintain the same notation regarding applicability of factors in partial fact situations: we write X⊨p when $X(p) = \textbf{t}$, and $X \vDash \lnot p$ when $X(p) = \textbf{f}$. In addition, we write $X \vDash {?p}$ to denote the expression $p \not \in {{\,\textrm{dom}\,}}(X)$, so $X \vDash {?p}$ holds if and only if *X* is undefined on *p*. Note that for partial fact situations the equivalence  no longer holds. In turn, we can now consider cases (*X*, *s*) where *X* is a partial fact situation, which represents a decision made by the decision-maker on the basis of incomplete information. We will say a case is partial, or complete, if its underlying fact situation is.

The use of partial fact situations and cases raises the question if, and if so how, Definition 1 of constraint should be altered. Suppose *Y* is a partial fact situation which was decided for side *s*, so (*Y*, *s*) is a partial case, when does the decision (*Y*, *s*) induce constraint on a partial focus fact situation *X*? Our philosophy when it comes to constraint induced by a partial case can be summarized as follows: if the status of a factor in the precedent case added strength to the side for which the case was decided, then the status of that factor should be matched in the focus fact situation in order for constraint to apply. We note three consequences of this view in particular:

- Factors on which the precedent case is undefined are inconsequential to the notion of constraint. We interpret a decision of *Y* for *s* as meaning that the factors in ${{\,\textrm{dom}\,}}(Y)$ were sufficient to arrive at a decision for *s*, and so only the factors in ${{\,\textrm{dom}\,}}(Y)$ should have an influence on the constraint induced by (*Y*, *s*).
- If *p* is a pro-*s* factor such that Y⊨p, then *p* added strength to the side *s* and so it should also apply in the focus fact situation in order for it to be said to have equal-or-greater support for side *s*. In particular, if *X* is undecided on *p* then (*Y*, *s*) should not induce constraint on *X* (just as in the case that *p* were false).
- Similarly, if *p* is a con-*s* factor such that $Y \vDash \lnot p$, then the status of *p* added support for *s*, and so it should be matched by *X*. In particular, if *X* is undecided on *p* then no constraint should be induced (just as in the case that *p* were true).

In principle, Definition 1 can be interpreted without problems when the focus fact situation *X* and the precedent case (*Y*, *s*) it involves are partial. However, in light of the aforementioned considerations, the resulting notion of constraint does not align with our intuitive notion of how constraint should work. The misalignment arises when we consider a con-*s* factor *p*, as illustrated in Table 1. The condition in Definition 1 for such a factor states that, in order for (*Y*, *s*) to induce constraint, the implication “if X⊨p then Y⊨p” should hold. In particular, when X⊨p and $Y \vDash {?}p$ then this implication does not hold, whereas intuitively we do not want to exclude constraint in such a scenario. Similarly, when $X \vDash {?}p$ and $Y \vDash \lnot p$ then constraint may still possibly be induced, while intuitively we want to exclude it. The rest of the possible scenarios do align with our intuition.

**Table 1:**
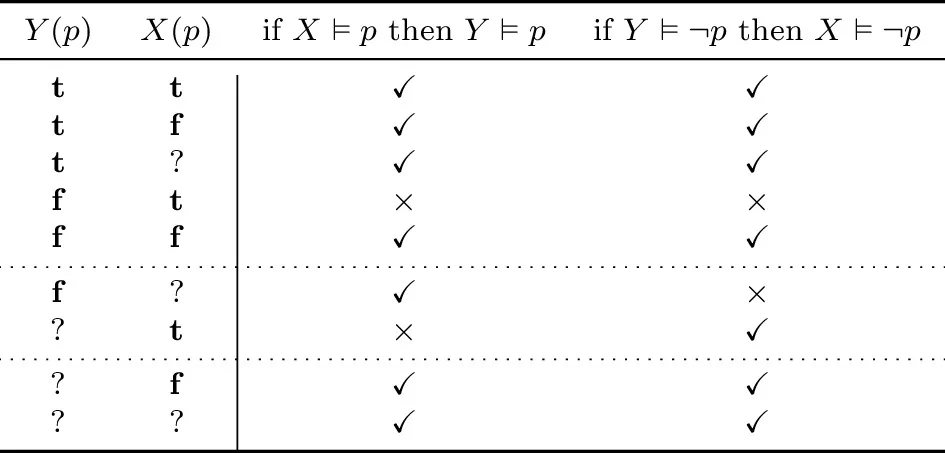
An overview of the differences for two possible prerequisites for a partial precedent case (*Y*, *s*) to induce constraint on a partial fact situation *X*. Here *p* is an arbitrary con-*s* factor, and every row represents a possible value assignment by the fact situations *X* and *Y*

The solution we thus propose is to instead use the implication “if $Y \vDash \lnot p$ then $X \vDash \lnot p$” for the con factors. As shown in Table 1 this does align with our intuition. In sum, this results in the following definition of constraint for partial fact situations.^3^

#### Definition 3

Let *X* be a partial fact situation and $\mathcal {C}$ a case base of partial cases, then *the decision of X* *for* π *is forced by* $\mathcal {C}$, denoted $\mathcal {C}, X \vDash \pi$, if and only if there is a case $(Y, \pi ) \in \mathcal {C}$ such that:

- for all $p \in {{\,\textrm{Pro}\,}}$: if Y⊨p then X⊨p, and
- for all $p \in {{\,\textrm{Con}\,}}$: if $Y \vDash \lnot p$ then $X \vDash \lnot p$.

Likewise, *the decision of X* *for* δ *is forced by* $\mathcal {C}$, denoted $\mathcal {C}, X \vDash \delta$ iff there is a case $(Y, \delta ) \in \mathcal {C}$ such that:

- for all $p \in {{\,\textrm{Pro}\,}}$: if $Y \vDash \lnot p$ then $X \vDash \lnot p$, and
- for all $p \in {{\,\textrm{Con}\,}}$: if Y⊨p then X⊨p.

Note that for complete fact situations the implication for con factors is just the contrapositive of the one used in Definition 1:This means that for complete fact situations Definitions 1 and 3 coincide, which allows us to use the same notation for both definitions.

#### Remark 4

As mentioned, a second version of Definition 3 is obtained by using the implication “if X⊨p then Y⊨p” for the con-*s* factors. For the sake of comparison we will refer to this version as option (2) of the definition, and the current version—using the implication “if $Y \vDash \lnot p$ then $X \vDash \lnot p$”—as option (1).

#### Example 5

We reconsider Example 2 with respect to Definition 3. Suppose it is not known whether *Y* is married or not, so $Y \vDash {?}{\textbf {Married}}$, but that *Y* was nevertheless deemed to be a high recidivism risk, so we have a precedent case (*Y*, 1). Should this case be able to induce constraint on a new fact situation *X* satisfying $X \vDash {\textbf {Married}}$? According to Definition 3 the answer is yes—since , there is no further requirement placed on *X* with regards to the ${\textbf {Married}}$ factor. In this regard, the situation is identical to the scenario in which *Y* *were* in fact married, i.e. it is as though we assume that $Y \vDash {\textbf {Married}}$. Similarly, suppose that the age of *X* is unknown, whilst it is known that *Y* is younger than 21; so $X \vDash {?}{\textbf {Age}}$ and $Y \vDash \lnot {\textbf {Age}}$. Again we ask: should (*Y*, 1) be able to constrain a decision for *X*? This time the answer is no—since $Y \vDash \lnot {\textbf {Age}}$ is a reason to decide *Y* for 1, the fact that $X \vDash {?}{\textbf {Age}}$ means that *X* does not match this support for a high-risk judgement and so constraint is excluded. In this regard, the situation is identical to the scenario in which *X* *were not* over the age of 21, i.e. it is as though we assume that $X \vDash {\textbf {Age}}$. These observations with regards to assumptions about the truth values of the undefined factors in the precedent and focus fact situations are part of a general pattern, to which we will return in Sect. 3.5.

### Monotonicity

Since the notion of constraint can be thought of as a type of entailment relation we can consider whether it is *monotonic*—can deductions be invalidated by the addition of information? Classical logic is well-known to be monotonic, which is formally captured by the implication that if $\Phi \vdash \phi$ and $\Phi \subseteq \Phi '$ then $\Phi ' \vdash \phi$, for any formula ϕ and sets of formulas $\Phi , \Phi '$. This property runs contrary to everyday reasoning, and so the monotonicity of classical logic has spurred much research on nonmonotonic logic and defeasible reasoning in general (Koons 2017; Strasser and Antonelli 2019).

One relevant adaptation of the statement of monotonicity for the rm is the question whether the forcing of a decision of a fact situation for a particular side can be invalidated by an expansion of the case base. It is not hard to see that the rm is indeed monotonic in this sense, as the following proposition shows.

#### Proposition 6

If $\mathcal {C}, X \vDash s$ and $\mathcal {C}\subseteq \mathcal {D}$ then $\mathcal {D}, X \vDash s$.

#### Proof

To show $\mathcal {D}, X \vDash s$ we need $(Y, s) \in \mathcal {D}$ satisfying the appropriate conditions of Definition 3. Since $\mathcal {C}, X \vDash s$ there is such a $(Y, s) \in \mathcal {C}$, and therefore by $\mathcal {C}\subseteq \mathcal {D}$ we have $(Y, s) \in \mathcal {D}$ as desired. □

However, the use of partial fact situations introduces a second form of monotonicity for consideration: can deductions be invalidated by adding information to the focus fact situation? We define the addition of information in this case using the notion of function extension.

#### Definition 7

Given a function f:A→B and a subset C⊆A we define the *restriction* $f{\upharpoonright }_{C}: C \rightarrow B$ of *f* to *C* by $f{\upharpoonright }_{C}(x) = f(x)$ for x∈C.

#### Definition 8

A function *f* is an *extension* of a function *g*, denoted by g⊆f, if ${{\,\textrm{dom}\,}}(g) \subseteq {{\,\textrm{dom}\,}}(f)$ and $f{\upharpoonright }_{{{\,\textrm{dom}\,}}(g)} = g$.

Since we consider fact situations to be just functions, these concepts are directly applicable to fact situations. In this context, a fact situation *Y* extends a fact situation *X* if they agree on all factors in ${{\,\textrm{dom}\,}}(X)$, but where *Y* is possibly defined on more factors than *X*. So, we can regard an extension *Y* of a fact situation *X* as containing more information than *X*, and ask whether the notion of constraint is monotonic with respect to the addition of this information. Again, this is indeed the case.

#### Proposition 9

Let *X* and *Y* be fact situations such that X⊆Y, then $\mathcal {C}, X \vDash s$ implies $\mathcal {C}, Y \vDash s$ for $s \in \{\pi , \delta \}$.

#### Proof

We consider the case where s=π, the other case is similar. Suppose $\mathcal {C}, X \vDash \pi$, then there is $(Z, \pi ) \in \mathcal {C}$ such that for all $p \in {{\,\textrm{Pro}\,}}$: Z⊨p implies X⊨p; and for all $p \in {{\,\textrm{Con}\,}}$: $Z \vDash \lnot p$ implies $X \vDash \lnot p$. We show that the same implications hold for *Y*, so that $\mathcal {C}, Y \vDash \pi$ as desired. Note that since, X⊆Y, X⊨p implies Y⊨p, and hence Z⊨p implies X⊨p implies Y⊨p, for any $p \in {{\,\textrm{Pro}\,}}$; the case for $p \in {{\,\textrm{Con}\,}}$ is analogous. □

#### Remark 10

The phrasing of Proposition 9 relies on the use of partial fact situations; if a fact situation is defined on all values, meaning it assigns true or false to each factor, then there is no way in which additional information can be added to it. It may be argued that there is a difference here with respect to the representation of fact situations as subsets of *F*, as used by Horty (2011), where a fact situation *X* is given as a subset X⊆F, so that the elements of *X* denote the factors that are deemed to apply in the situation represented by *X*. We could then ask whether $\mathcal {C}, X \vDash s$ and X⊆Y implies $\mathcal {C}, Y \vDash s$, and indeed this implication does not hold. However, Horty (2011) does not specify the meaning of the absence of a factor from a fact situation, so $p \not \in X$ could mean either that *p* explicitly does not hold in *X*, or that it is unknown whether *p* applies in *X*. In the former case, the addition of *p* to *X* does not constitute an addition, but rather a modification of information to *X*—the ‘truth value’ of *p* is changed from false to true. This would not align with the usual interpretation of monotonicity in logic; for example, $\Phi \cup \{\lnot p\} \vdash \phi$ does not imply $\Phi \cup \{p\} \vdash \phi$ in propositional logic. Therefore, we feel this further distinction, made by partiality of fact situations, is necessary in the context of a discussion about monotonicity in the addition of information to fact situations.

#### Remark 11

It is interesting to note that for option (2) of the definition of constraint (see Remark 4), the notion of constraint is still monotonic in the case base, but *non*monotonic with respect to the addition of information to fact situations. This can be understood by looking at the row in Table 1 corresponding to the scenario where *X*(*p*) is undefined, and $Y \vDash \lnot p$. With option (2), constraint is not excluded in this scenario. Extending the fact situation *X* to *Z* (so X⊆Z) with Z⊨p would mean constraint is excluded, as illustrated by the fourth row in the table. This pattern, where options (1) and (2) give different results with respect to monotonicity of constraint, continues in the hierarchical version of the result model which we introduce in the next section; cf. Remark 27.

As an application of monotonicity, we will now formalize the observation made in Example 5 with regards to our treatment of undefined factors in Definition 3 of constraint, which can be phrased informally as follows. Given an outcome *s* and a precedent case (*Y*, *s*), the pro-*s* factors that are undefined in *Y* are treated as if they were false, and the con-*s* factors that are undefined in *Y* are treated as if they were true. Conversely, in a focus fact situation *X* the pro-*s* factors that are undefined in *X* are treated as if they were true, and the con-*s* factors are treated as if they were false. That this is true can be partially read off from Table 1: the rows in which *Y*(*p*) is undefined are identical to the rows in which *Y*(*p*) is false (with respect to the condition “if $Y\vDash \lnot p$ then $X \vDash \lnot p$”), and the same holds for *X*(*p*). We will now phrase and prove this formally.

Let *X* be a fact situation, $s \in \{\pi , \delta \}$ an outcome, and define a fact situation $\underline{X}_s$ by:

**Figure:**
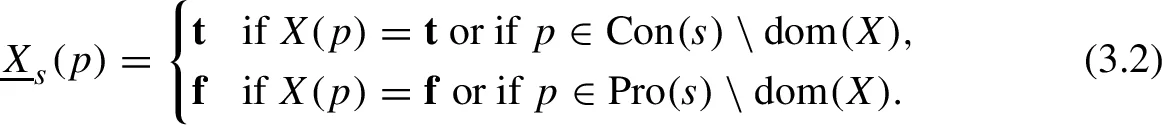

Note that $X \subseteq \underline{X}_s$ and that $\underline{X}_s$ is complete, meaning ${{\,\textrm{dom}\,}}(\underline{X}_s) = F$. We start with a lemma.

#### Lemma 12

For $p \in {{\,\textrm{Pro}\,}}(s)$, if $\underline{X}_s \vDash p$ then X⊨p; for $p \in {{\,\textrm{Con}\,}}(s)$, if $\underline{X}_s \vDash \lnot p$ then $X \vDash \lnot p$.

#### Proof

Let $p \in {{\,\textrm{Pro}\,}}(s)$ and suppose $\underline{X}_s \vDash p$. By () this means that either X⊨p or $p \in {{\,\textrm{Con}\,}}(s)\setminus {{\,\textrm{dom}\,}}(X)$. This latter option contradicts the assumption that $p \in {{\,\textrm{Pro}\,}}(s)$, and so indeed X⊨p. The reasoning for the second implication follows the same pattern. □

Let *X* and *Y* be some fact situations. We have the following propositions.

#### Proposition 13

$\{(Y, s)\}, X \vDash s$ if and only if $\{(\underline{Y}_s, s)\}, X \vDash s$.

#### Proof

For the left-to-right direction, we assume that $\{(Y, s)\}, X \vDash s$. Now, note that by Lemma 12 we have that for all $p \in {{\,\textrm{Pro}\,}}(s)$, $\underline{Y}_s \vDash p$ implies Y⊨p, and so X⊨p by the assumption that $\{(Y, s)\}, X \vDash s$. In the same vein Lemma 12 shows that for all $p \in {{\,\textrm{Con}\,}}(s)$, $\underline{Y}_s \vDash \lnot p$ implies $X \vDash \lnot p$ and so indeed $\{(\underline{Y}_s, s)\}, X \vDash s$. The right-to-left direction follows readily from $Y \subseteq \underline{Y}_s$; for example if $p \in {{\,\textrm{Pro}\,}}(s)$, then Y⊨p implies $\underline{Y}_s \vDash p$ and so X⊨p by assumption. □

#### Proposition 14

$\{(Y, s)\}, X \vDash s$ if and only if $\{(Y, s)\}, \underline{X}_s \vDash s$.

#### Proof

Left-to-right is an instance of Proposition 9. For the other direction, suppose that $\{(Y, s)\}, \underline{X}_s \vDash s$. Now if $p \in {{\,\textrm{Pro}\,}}(s)$ and Y⊨p, then $\underline{X}_s \vDash p$ by assumption and so X⊨p by Lemma 12. The case for $p \in {{\,\textrm{Con}\,}}(s)$ is similar, and so we indeed have $\{(Y, s)\}, X \vDash s$. □

Lastly, we show that Propositions 13 and 14 also hold for case bases. To this end, let $\mathcal {C}$ be a case base, and define $\underline{\mathcal {C}} = \{(\underline{Y}_s, s) \mid (Y, s) \in \mathcal {C}\}$.

#### Theorem 15

$\mathcal {C}, X \vDash s$ if and only if $\underline{\mathcal {C}}, \underline{X}_s \vDash s$.

#### Proof

For left-to-right, suppose that $\mathcal {C}, X \vDash s$; then there is some $(Y, s) \in \mathcal {C}$ such that $\{(Y, s)\}, X \vDash s$. By Propositions 13 and 14 we thus have $\{(\underline{Y}_s, s)\}, \underline{X}_s \vDash s$, and so since $\{(\underline{Y}_s, s)\} \subseteq \underline{\mathcal {C}}$ we get $\underline{\mathcal {C}}, \underline{X}_s \vDash s$ from Proposition 6. The other direction is analogous. □

Theorem 15 shows that any instance of partial constraint can be understood as an instance of complete constraint, in which pro-*s* factors are taken to be false, and undefined con-*s* factors are taken to be true. In this sense, our approach to partial constraint can be seen as assigning *default* truth values to undefined factors, relative to an outcome *s*. These default truth values are the “worst-case” values with respect to the outcome *s*—undefined con-*s* factors are assumed to be true, and pro-*s* factors are assumed to be false. This is comparable to the recently proposed approach to partial fact situations and partial cases by Odekerken et al. (2024). In particular, the notion of STABLE-*s* forcing of Odekerken et al. (2024) is comparable to the default truth value description of our proposed definition of forcing for partial fact situations, but a complete formal comparison between these two definitions is beyond the scope of this work.

#### Example 16

We turn once more to the set of recidivism factors in (3.1). Suppose *Y* was a fact situation with $Y \vDash \{{?}{\textbf {Record}}, {\textbf {Male}}, \lnot {\textbf {Education}}, {?}{\textbf {Married}}, \lnot {\textbf {Age}}\}$. Furthermore, suppose *Y* was deemed to pose a recidivism risk, so that we have a case (*Y*, 1). Proposition 13 tells us *Y* induces the same constraint as its completion $(\underline{Y}_1, 1)$. Applying the definition in () we see that $\underline{Y}_1 \vDash \{\lnot {\textbf {Record}}, {\textbf {Married}}\}$. In other words, our proposed definition of constraint treats (*Y*, 1) as if *Y* assigned true to the ${\textbf {Married}}$ factor, and false to the ${\textbf {Record}}$ factor. The same holds for a focus fact situation *X* satisfying, for example, $X \vDash \{{?}{\textbf {Record}}, \lnot {\textbf {Male}}, {\textbf {Education}}, {?}{\textbf {Married}}, {\textbf {Age}}\}$; Proposition 14 states that *X* is forced for outcome 1 by a precedent case if and only if $\underline{X}_1$ is forced by that precedent case, and $\underline{X}_1 \vDash \{\lnot {\textbf {Record}}, {\textbf {Married}}\}$. Note, however, that this situation is reversed with respect to constraint for outcome 0: we then have $\underline{X}_0 \vDash \{{\textbf {Record}}, \lnot {\textbf {Married}}\}$.

#### Remark 17

For option (2) of Definition 3 a similar result holds, but then both pro-*s* and con-*s* are interpreted as being false, rather than just the pro-*s* factors.

## The hierarchical result model

The goal of this section is to develop a version of the rm that accommodates additional hierarchical structure on the set of factors, which we do based on ideas in earlier work on formal models of precedential constraint by Roth (2003); Roth and Verheij (2004).

We begin in Sect. 4.1 by giving an example of a factor hierarchy, based on the running example introduced in Sect. 3.1, and then formalize the notion of factor hierarchy as a knowledge representation in Sect. 4.2. We then discuss some possible approaches to adapting the notion of constraint from the rm to this extended representation in Sect. 4.3, and subsequently present our variant in Sect. 4.4. We refer to the resulting model as the hrm. The remainder of the sections is dedicated to analyzing formal properties of this definition. In Sect. 4.5 we show the hrm is monotonic in the addition of cases and in the addition of information to a focus fact situation. We conclude in Sect. 4.6 by showing that the rm is a special case of the hrm, or in other words, that the hrm is a conservative extension of the rm.

### An example of a factor hierarchy

A downstream purpose of recidivism risk assessment, and an example of the purposes for which the compas program is used in practice, is to determine whether a defendant should be released on bail. Bail is a sum of money that the defendant must pay to the court as a guarantee that they will appear at their trial—if the defendant does not appear, the bail is forfeited. The decision to grant bail, like recidivism risk, is influenced by several factors; e.g. a defendant with a high risk of flight is less likely to be granted bail, while one with a history of appearing to court is more likely to be granted bail.

In other words, determining bail is a domain to which the result model can be applied, but this time one of the input factors—risk of recidivism—can itself be determined on the basis of a fortiori reasoning. This situation is described as a *factor hierarchy* in the ai & law literature. The hierarchical structure of factors was first recognized by Aleven in his work on the cato program, and has since become a standard component of work in ai & law involving factor-based knowledge representations; see e.g. the work by Prakken and Sartor (1998); Roth (2003); Bruninghaus and Ashley (2003); Grabmair (2017), among others.

We expand example (3.1) from Sect. 3.1 to a factor hierarchy for a bail decision:

**Figure:**
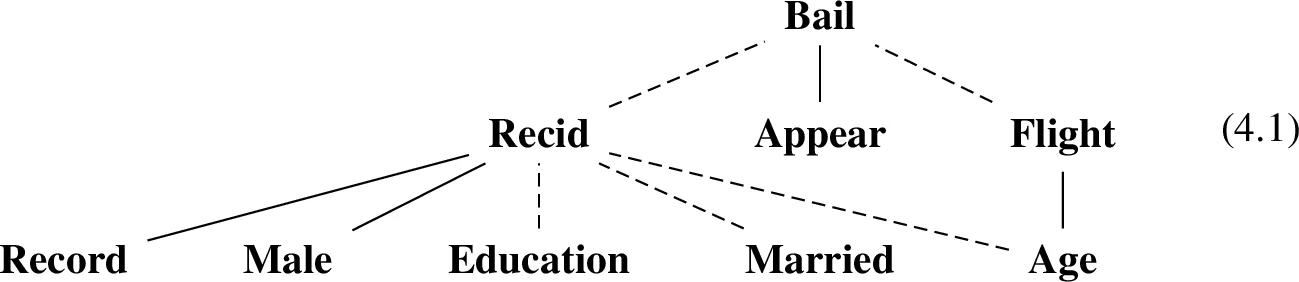

The ${\textbf {Bail}}$ node corresponds to a decision to grant bail. The links are either solid or dotted, which carries the same meaning as it did in our earlier example; for instance, the dotted line from ${\textbf {Recid}}$ to ${\textbf {Bail}}$ indicates that high risk of recidivism suggests bail should be denied. Two other factors are added: ${\textbf {Appear}}$, which represents that the defendant has a history of always appearing for trial, and ${\textbf {Flight}}$, which stands for a low or high perceived risk of fleeing. This means that in this example we assume that older people are more likely to flee before trial than younger people. Whether this assumption holds in practice is debatable—it is added primarily to exemplify that factors can influence multiple higher level factors. Furthermore, the hierarchy is not intended to be an exhaustive representation of the real-world factors influencing a bail decision.

### Knowledge representation

We now expand the knowledge representation used by the rm to one incorporating hierarchical structure on the set *F* of factors. Horty (2011, Section 7) discusses a possible extension of his result and reason models of precedential constraint to account for hierarchical structure. He remarks that in such an extension, the outcomes π and δ can be taken as special factors, and a precedent case as “a linked set of precedent constituents, beginning with a characterization of the initial fact situation as a set of base-level factors, proceeding through a series of higher-level legal concepts, and eventually arriving at a decision [...] for the plaintiff or defendant.” This is very similar to the approach taken by Roth (2003), who represents cases as sets of factors and links between them (indicating the hierarchical structure), and in which the case outcome is considered to be just one of the factors.

We will use a slightly different approach in this work. Firstly, we will not assume that the set of factors necessarily contains a factor corresponding directly to the case outcome. This is to accommodate the point of view, as recently advocated by e.g. Bench-Capon and Atkinson (2021), that precedent cases serve to determine the applicability of legal *issues*—the legal questions that need to be answered in order to determine the outcome of the case. Proponents of this view argue that case outcomes are determined as a logical function of the legal issues of the domain, and not directly on the basis of precedent; rather, it is the applicability of the issues that is determined by precedent. In our approach, we assume that the factor hierarchy of the domain culminates in one or more factors, which may either correspond to the issues of the domain, or (if there is just one) directly to the case outcome. For the sake of discussion we will henceforth refer to the top-level elements of the hierarchy simply as issues, even in the case where there is a single top-level element corresponding directly to the case outcome.

Secondly, we will not consider the hierarchical structure to be part of individual cases—as in the representation used by Roth—but rather as a separate structure which the set of factors is endowed with. More specifically, we model this structure using a relation on the set *F* of factors.

#### Definition 18

A *factor hierarchy* $(F, \textsf{Pro}, \textsf{Con})$ consists of a finite set of factors *F* with two binary relations $\textsf{Pro}$ and $\textsf{Con}$ on *F*, such that $\textsf{Pro}\cap \textsf{Con}= \emptyset$, and that the transitive closure of their union $\textsf{H}= \textsf{Pro}\cup \textsf{Con}$ is irreflexive.

In other words, a factor hierarchy consists of a set of factors as in the $\textsc {rm}$, which is given hierarchical structure by a relation $\textsf{H}$; given factors p,q∈F the relation $\textsf{H}(p, q)$ means *p* is directly below *q* in the hierarchy. The hierarchical structure may not contain loops, and any link $\textsf{H}(p, q)$ represents either that the presence of *p* lends defeasible support to the presence of *q*—if $\textsf{Pro}(p, q)$—or that it lends defeasible opposition to the presence of *q*—if $\textsf{Con}(p, q)$—but never both, as $\textsf{Pro}\cap \textsf{Con}= \emptyset$.

All the usual terminology related to factor hierarchies can now be defined in terms of the structure $(F, \textsf{Pro}, \textsf{Con})$. An $\textsf{H}$-minimal factor is called *base-level*, and we write *B* for the set of all base-level factors in the hierarchy. A factor that is not base-level is called *abstract*, and the set of abstract factors is denoted by *A*, and so *F* is partitioned by F=A∪B. Factors are assumed to support or oppose each other in hierarchical fashion, as indicated by the relations $\textsf{Pro}$ and $\textsf{Con}$. When $\textsf{Pro}(p, q)$ holds we say *p* is a pro-*q* factor, and $\textsf{Con}(p, q)$ we say *p* is a con-*q* factor. Note that any subset G⊆F of the set of factors, together with $\textsf{H}$ restricted to *G*, yields a factor hierarchy $(G, \textsf{Pro}, \textsf{Con})$.

In the rm, a fact situation is made a case by pairing it with an outcome. In Definition 18 of a factor hierarchy, the distinction between factors and issues, or case outcomes, is dropped. This means that a complete fact situation now contains a decision on all issues, or on the case outcome. Therefore, to be able to distinguish between fact situations that have been decided on the issues of the hierarchy and those that have not, we also drop the requirement that a fact situation is defined on all factors. More specifically, a fact situation *X* is now a valuation of a subset of *F*, i.e., a partial fact situation as before, and again we write ${{\,\textrm{dom}\,}}(X)$ for the subset of *F* on which *X* is defined. Similarly, we write X⊨p to mean that $X(p) = \textbf{t}$. A difference with the rm is that *p* may now be an abstract factor, such as an issue, in which case the meaning of X⊨p is that it was *decided* by the decision-maker that *p* applies in *X*. In general, the idea is these decisions are made on the basis of a balancing of the pro- and con-*p* factors, possibly coupled with precedential reasoning, such as when the decision-maker is a court. However, our formalism does not make explicit assumptions about the way in which the decision-maker reasons.

In this knowledge representation we also allow cases to be partial. The reason for this is twofold. Firstly, pragmatically, we want to allow the possibility for cases to be undefined on factors because in practice the decision-maker, such as a court, may not make a decision about every factor of the domain. Secondly, formally, no further requirements on cases are needed for our definition of constraint (cf. Sect. 4.3). This generality allows users of the hrm to further augment the definition of a case, if needed. For example, in practice it could be natural to require that a case—by definition—is decided on at least one of the issues of the factor hierarchy. Furthermore, as a second example of a natural requirement of cases, we could ask that for each abstract factor *p* which is assigned true in the case, at least one pro-*p* factor *q* is also assigned true in the case. However, we stress again that neither of these requirements is necessary for defining a notion of forcing in our framework, and so we omit them.

### Approaches to hierarchical constraint

Recognizing the hierarchical structure of factors poses questions about how this structure should influence the notion of constraint, which are a matter of ongoing debate in the literature. As an example to guide this discussion we will consider the penitentiary law domain. Much research has been done on the relevant factors in this domain. Below, we consider a decision of whether the defendant should be released on bail. The factors are taken from the work by Mamalian (2011) and Gouldin (2018).

**Figure:**
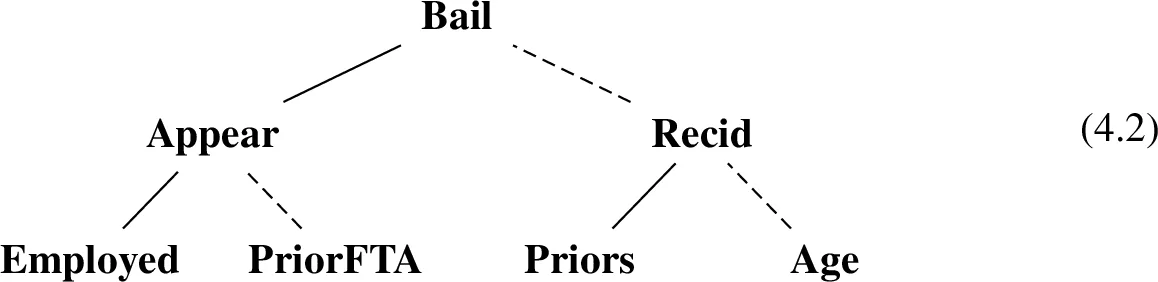

The first question we consider is whether constraint should work only on the issues of the hierarchy, or more generally on all abstract factors. For example, in the hierarchy depicted by (4.2), should previous decisions about the recidivism risk factor ${\textbf {Recid}}$ constrain future decisions about this factor? Or should constraint be limited to function only on the ultimate decision about granting bail, represented by the ${\textbf {Bail}}$ factor? Bench-Capon (2023) argues that the abstract factors appearing in factor hierarchies in the literature do not form part of the law in the domain they are describing; therefore, there is no suggestion that judges would acknowledge them. In this view, decisions about abstract factors should not induce constraint, at least in the legal setting. In related work on hierarchical constraint, Canavotto and Horty (2023a) argue in response to Bench-Capon’s objections that formal work on hierarchical constraint describes normative reasoning more generally, and have wider potential applicability than just to the domain of law. Indeed, the notion of hierarchical constraint put forth by  Canavotto and Horty (2023b, Definition 9) functions on the abstract factors, in addition to the issues of a factor hierarchy. We agree with this more general view, and will define constraint to operate on abstract factors.

The second question we consider is whether the status of the abstract factors of a precedent case should play a role in the constraint that it induces, or whether it should be only the status of the base-level factors that influence whether constraint is applied. In other words, given a fact situation *X* and an abstract factor *p*, should changing the truth values in *X* of the abstract factors below *p* have an influence on the constraint induced by *X* with respect to *p*? The definition of constraint developed by Roth (2003); Roth and Verheij (2004) takes this second stance: Constraint is determined entirely by the truth status of the base-level factors. Following terminology of Canavotto and Horty (2023b, 2023a), we will refer to this as *flat* constraint. This involves successively adding connections to the hierarchy from lower level factors to higher level ones, in order to ultimately be able to say whether the base-level factors support or oppose abstract factors that they are not directly connected to in the hierarchy. This then gives rise to an instance of the regular rm, so that its notion of constraint can be applied as before.

To exemplify Roth’s (2003, Definition 6) approach, a form of which can also be found in the work by Canavotto and Horty (2023b, Definition 2), we consider the factor hierarchy depicted in (4.2). Since the base-level factors in this hierarchy do not directly connect to the bail decision issue, we can not apply the rm as is. However, there are (by definition) paths in the hierarchy from the base-level factors leading up to the issues. The idea of flat constraint is to successively add missing links on the basis of the polarity of these intermediate links. For instance, in (4.2), the base-level factors do not have links directly to the **Bail** issue, but do have links to the intermediate abstract factors **Appear** and **Recid**. Since **Employed** supports the abstract factor **Appear**, which in turn supports the **Bail** issue, we can see that the presence of the **Employed** factor also supports a decision to grant bail. Therefore, we could extend the hierarchy to include a direct link of support from the **Employed** factor to the **Bail** factor. Similarly, the **Priors** factor is a pro-**Recid** factor, which is itself a con-**Bail** factor. So, we can conclude that prior offenses indirectly suggest bail should not be granted, which can be encoded from a negative link from **Priors** to **Bail**. In the end, we obtain:

**Figure:**
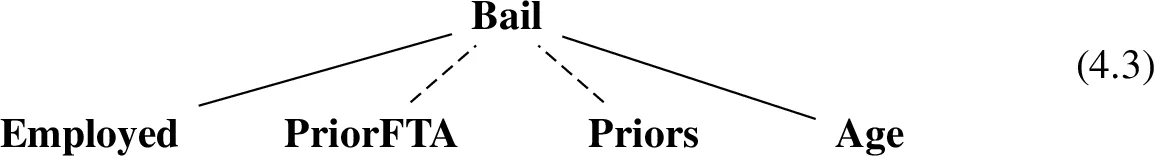

This flattened hierarchy constitutes a factor partition as we discussed in Sect. 3.2, and so the notion of constraint of the rm can be applied here. We see that in the resulting notion of constraint, the status of the intermediate factors in the constraining precedent case becomes inconsequential.

An alternative to flat constraint is what is called *hierarchical* constraint by Canavotto and Horty (2023b, 2023a). In this approach, the constraint is induced directly on the basis of the status of the abstract factors in the precedent case. The idea underlying this approach is that it is the status of the factors that is consequential (with respect to connected higher level factors), rather than the reason for their status (resulting by weighing of connected lower level factors). For example, looking again at (4.2), this approach argues that when it comes to a decision about granting bail, what matters more is whether someone is at risk to recidivate, rather than why someone is at risk to recidivate.

In the subsequent section we implement hierarchical constraint by use of recursion. Each abstract factor *p* in the hierarchy, together with its direct subordinate pro- and con-*p* factors, is taken as an instance of the regular rm. Constraint is then applied recursively, each step involving the citation of a precedent case. This allows for the notion of constraint to involve several precedent cases, successively building up constraint on the more abstract levels of the hierarchy. To illustrate, we again consider the factor hierarchy depicted in (4.2). Suppose there is a precedent case $c_1$ in which it was determined that a defendant over the age of 21 with prior convictions was deemed at high risk of recidivism. In other words, the **Priors** factor outweighs the **Age** factor when it comes to determining risk of recidivism. Furthermore, suppose there is a second precedent case $c_2$ in which it was determined that a high risk of recidivism is reason enough to deny bail, even when the defendant has a high chance of appearing to court. This means it was determined that the **Recid** factor outweighs the **Appear** factor. In the presence of these two precedent cases, any fact situation containing the **Priors** factors should therefore be forced for the outcome of having bail denied: first $c_1$ constrains the decision for a high recidivism risk judgement, and then, on the basis of this high recidivism risk, $c_2$ constrains a decision for bail denial.

### The recursive approach to constraint

To define constraint we write $\textsf{Pro}(p)$ for the pre-image of *p* under the relation $\textsf{Pro}$, so $\textsf{Pro}(p) = \{q \in F \mid \textsf{Pro}(q, p)\}$, and similarly $\textsf{Con}(p)$ for its pre-image under $\textsf{Con}$. We now define two relations $\mathcal {C}, X \vDash p$ and $\mathcal {C}, X \vDash \lnot p$ of constraint for the hrm by mutual recursion.

#### Definition 19

Let p∈F, *X* a fact situation and $\mathcal {C}$ a case base, then *the decision of X* *for p* *is forced by* $\mathcal {C}$, denoted $\mathcal {C}, X \vDash p$, if and only if either

- X⊨p, or
- p∈A and there is a case $Y \in \mathcal {C}$ with Y⊨p and
  - for all $q \in \textsf{Pro}(p)$: if Y⊨q then $\mathcal {C}, X \vDash q$, and
  - for all $q \in \textsf{Con}(p)$: if $Y \vDash \lnot q$ then $\mathcal {C}, X \vDash \lnot q$.

Likewise, *the decision of X* *for* ¬p *is forced by* $\mathcal {C}$, denoted $\mathcal {C}, X \vDash \lnot p$ iff either

- $$X \vDash \lnot p$$, or
- p∈A and there is a case $Y \in \mathcal {C}$ with $Y \vDash \lnot p$ and
  - for all $q \in \textsf{Pro}(p)$: if $Y \vDash \lnot q$ then $\mathcal {C}, X \vDash \lnot q$, and
  - for all $q \in \textsf{Con}(p)$: if Y⊨q then $\mathcal {C}, X \vDash q$.

#### Remark 20

Definition 19 of constraint differs from that proposed by van Woerkom et al. (2023b), who use the implication “if $\mathcal {C}, X \vDash q$ then Y⊨q” for the con-*p* factors *q*, whereas Definition 19 uses the implication “if $Y \vDash \lnot q$ then $\mathcal {C}, X \vDash \lnot q$”. This is entirely analogous to the differences discussed in Sect. 3.4 and corresponds to the two options (1) and (2) discussed in Remark 4.

#### Remark 21

Note that, according to this definition of constraint, in order for a fact situation *X* to induce constraint on an abstract factor *p* we should have $p \in {{\,\textrm{dom}\,}}(X)$, meaning *X* should be defined on *p*. Therefore, in order to serve as a precedent in the usual sense of the word, a fact situation *X* should be defined on at least one abstract factor.

We note two simple consequences of this definition. Firstly, any factor p∈F that is deemed to hold in a fact situation *X*, so X⊨p, continues to hold in the presence of a case base $\mathcal {C}$.

#### Lemma 22

If X⊨p then $\mathcal {C}, X \vDash p$. Likewise, $X \vDash \lnot p$ implies $\mathcal {C}, X \vDash \lnot p$.

For base-level factors—i.e. those in B=F\A—the converse of Lemma 22 holds too.

#### Lemma 23

If p∈B then $\mathcal {C}, X \vDash p$ implies X⊨p, and $\mathcal {C}, X \vDash \lnot p$ implies $X \vDash \lnot p$.

#### Proof

As p∈B we have $p \not \in A$, and so spelling out Definition 19 gives:

$$\begin{aligned}&\mathcal {C}, X \vDash p \\&\text {iff }\bullet X \vDash p, \text { or} \\&\quad \bullet {p \in A} \text { and there is }Y \in \mathcal {C}\text { with } Y \vDash p \text { and} \\&\qquad - \text { for all } q \in \textsf{Pro}(p)\!\!: \text { if } W \vDash q \text { then } \mathcal {C}, X \vDash q, \text { and} \\&\qquad - \text { for all } q \in \textsf{Con}(p)\!\!: \text { if } W \vDash \lnot q \text { then } \mathcal {C}, X \vDash \lnot q \\&\text {iff } X \vDash p. \end{aligned}$$

□

#### Example 24

To illustrate Definition 19 we work through an example based on the factor hierarchy depicted in (4.1), which is formally specified by $(F, \textsf{Pro}, \textsf{Con})$ where

$$\begin{aligned} F = \{&{\textbf {Bail}}, {\textbf {Recid}}, {\textbf {Appear}}, {\textbf {Flight}}, {\textbf {Record}}, {\textbf {Male}}, {\textbf {Education}}, {\textbf {Married}}, {\textbf {Age}}\}, \\ \textsf{Pro}= \{&({\textbf {Record}}, {\textbf {Recid}}), ({\textbf {Male}}, {\textbf {Recid}}), ({\textbf {Appear}}, {\textbf {Bail}}), ({\textbf {Age}}, {\textbf {Flight}})\}, \\ \textsf{Con}= \{&({\textbf {Education}}, {\textbf {Recid}}), ({\textbf {Married}},{\textbf {Recid}}), ({\textbf {Age}}, {\textbf {Recid}}), ({\textbf {Recid}}, {\textbf {Bail}}), \\&({\textbf {Flight}}, {\textbf {Bail}})\}. \end{aligned}$$

Let *X* and *Y* be two fact situations for this hierarchy, defined by

$$\begin{aligned} X&\vDash \{?{\textbf {Bail}}, ?{\textbf {Recid}}, {\textbf {Appear}}, {\textbf {Flight}}, {\textbf {Record}}, \lnot {\textbf {Male}}, {\textbf {Education}}, \lnot {\textbf {Married}}, {\textbf {Age}}\}, \\ Y&\vDash \{{\textbf {Bail}}, \lnot {\textbf {Recid}}, {\textbf {Appear}}, {\textbf {Flight}}, \lnot {\textbf {Record}}, {\textbf {Male}}, {\textbf {Education}}, {\textbf {Married}},\lnot {\textbf {Age}}\}. \end{aligned}$$

We consider whether the decision to grant bail to the defendant in fact situation *Y* induces constraint for the decision to grant bail to the defendant in the focus fact situation *X*:

$$\begin{aligned}&\{Y\}, X \vDash {\textbf {Bail}}\\ \text {iff }&\bullet X \vDash {\textbf {Bail}}, \text { or} \\&\bullet {\textbf {Bail}}\in A \text { and there is } W \in \{Y\} \text { with } W \vDash {\textbf {Bail}}\text { and} \\&\quad -\text { for all } q \in \textsf{Pro}({\textbf {Bail}})\!\!: \text { if } W \vDash q \text { then } \{Y\}, X \vDash q, \text { and} \\&\quad - \text { for all } q \in \textsf{Con}({\textbf {Bail}})\!\!: \text { if } W \vDash \lnot q \text { then } \{Y\}, X \vDash \lnot q \\ \text {iff }&\{Y\}, X \vDash {\textbf {Appear}}\text { and } \{Y\}, X \vDash \lnot {\textbf {Recid}}. \end{aligned}$$

As $X \vDash {\textbf {Appear}}$, and so $\{Y\}, X \vDash {\textbf {Appear}}$, this simplifies to $\{Y\}, X \vDash \lnot {\textbf {Recid}}$, but

$$\begin{aligned}&\{Y\}, X \vDash \lnot {\textbf {Recid}}\\ \text {iff }&\bullet X \vDash \lnot {\textbf {Recid}}, \text { or} \\&\bullet {\textbf {Recid}}\in A \text { and there is } W \in \{Y\} \text { with } W \vDash \lnot {\textbf {Recid}}\text { and} \\&\quad -\text { for all }q \in \textsf{Pro}({\textbf {Recid}})\!\!: \text { if } W \vDash \lnot q \text { then }\{Y\}, X \vDash \lnot q, \text { and} \\&\quad -\text { for all }q \in \textsf{Con}({\textbf {Recid}})\!\!: \text { if } W \vDash q \text { then }\{Y\}, X \vDash q \\ \text {iff }&\{Y\}, X \vDash \lnot {\textbf {Record}}, \{Y\}, X \vDash {\textbf {Married}}, \text { and }\{Y\}, X \vDash {\textbf {Education}}, \\ \text {iff }&X \vDash \{\lnot {\textbf {Record}}, {\textbf {Married}}, {\textbf {Education}}\}. \end{aligned}$$

The last equivalence follows from Lemma 23. By definition $X \vDash {\textbf {Record}}$ and so  and thus , meaning there is no constraint induced by *Y*.

The case *Y* does not constrain *X* in this example, because in *Y* it was decided that the defendant was not at risk of recidivism while *X* is undecided on this factor. Furthermore, *Y* does not force a low risk of recidivism assessment in *X*. However, due to the recursive call in Definition 19, a second precedent case may be used to force a decision in *X* for $\lnot {\textbf {Recid}}$. To illustrate this, we now supplement the case base $\{Y\}$ with a case *Z* such that

$$\begin{aligned}Z \vDash \{\lnot {\textbf {Recid}}, {\textbf {Record}}, \lnot {\textbf {Male}}, {\textbf {Education}}, \lnot {\textbf {Married}}, \lnot {\textbf {Age}}\}.\end{aligned}$$

This new case base $\{Y, Z\}$ does constrain *X* on the recidivism factor:

$$\begin{aligned}&\{Y, Z\}, X \vDash \lnot {\textbf {Recid}}\\ \text {iff }&\bullet X \vDash \lnot {\textbf {Recid}}, \text { or} \\&\bullet {\textbf {Recid}}\in A \text { and there is }W \in \{Y, Z\} \text { with }W \vDash \lnot {\textbf {Recid}}\text { and} \\&\quad -\text { for all }q \in \textsf{Pro}({\textbf {Recid}})\!\!: \text { if }W \vDash \lnot q \text { then }\{Y, Z\}, X \vDash \lnot q,\, \text {and} \\&\quad -\text { for all }q \in \textsf{Con}({\textbf {Recid}})\!\!: \text { if }W \vDash q \text { then }\{Y, Z\}, X \vDash q \\ \text {iff }&\{Y, Z\}, X \vDash \lnot {\textbf {Male}},\text { and }\{Y, Z\}, X \vDash {\textbf {Education}}, \\ \text {iff }&X \vDash \{\lnot {\textbf {Male}}, {\textbf {Education}}\}. \end{aligned}$$

We thus find that $\{Y, Z\}, X \vDash \lnot {\textbf {Recid}}$ holds. Furthermore, as $X \vDash {\textbf {Appear}}$ we have $\{Y, Z\}, X \vDash {\textbf {Appear}}$ by Lemma 22 and similarly $\{Y, Z\}, X \vDash {\textbf {Flight}}$ which means that $\{Y, Z\}, X \vDash {\textbf {Bail}}$ by the earlier line of reasoning.

### Monotonicity

The hrm, like the rm, is monotonic in the addition of new cases.

#### Proposition 25

Let p∈F, $\mathcal {C}\subseteq \mathcal {D}$ be two case bases, and *X* some fact situation; then $\mathcal {C}, X \vDash p$ implies $\mathcal {D}, X \vDash p$, and $\mathcal {C}, X \vDash \lnot p$ implies $\mathcal {D}, X \vDash \lnot p$.

#### Proof

We proceed by mutual induction on the position of *p* in the hierarchy $\textsf{H}$. For the base case, when *p* is $\textsf{H}$-minimal (so p∈B) Lemmas 22 and 23 tell us

$$\begin{aligned}\mathcal {C}, X \vDash p \quad \text {iff}\quad X \vDash p \quad \text {iff}\quad \mathcal {D}, X \vDash p,\end{aligned}$$

and similarly $\mathcal {C}, X \vDash \lnot p \text { iff } \mathcal {D}, X \vDash \lnot p$. In the induction case, when p∈A, we know that for all $q \in \textsf{Pro}(p) \cup \textsf{Con}(p)$: $\mathcal {C}, X \vDash q$ implies $\mathcal {D}, X \vDash q$, and $\mathcal {C}, X \vDash \lnot q$ implies $\mathcal {D}, X \vDash \lnot q$; therefore

**Figure:**
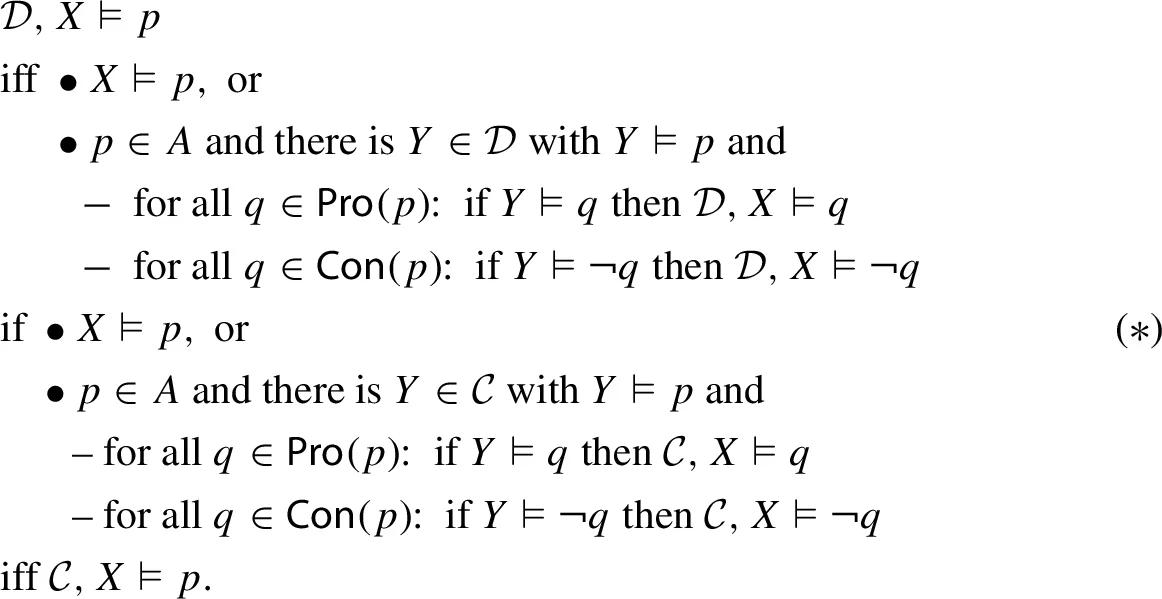

Step (∗) follows from $\mathcal {C}\subseteq \mathcal {D}$ and the induction hypothesis: if there is $Y \in \mathcal {C}$ satisfying the conditions described of (∗), then $Y \in \mathcal {D}$. Furthermore, given $q \in {{\,\textrm{Pro}\,}}(p)$, if Y⊨q, then $\mathcal {C}, X \vDash q$ and so $\mathcal {D}, X \vDash q$ by the induction hypothesis; the case for $q \in \textsf{Con}(p)$ follows the same pattern. By similar reasoning we can show $\mathcal {C}, X \vDash \lnot p$ implies $\mathcal {D}, X \vDash \lnot p$, which completes the induction case and thus the proof. □

Just as the rm, the hrm is also monotonic with respect to the extension of fact situations. The proof of this is just as in Proposition 9, so we omit it.

#### Proposition 26

If X⊆Y then $\mathcal {C}, X \vDash p$ implies $\mathcal {C}, Y \vDash p$, and $\mathcal {C}, X \vDash \lnot p$ implies $\mathcal {C}, Y \vDash \lnot p$.

#### Remark 27

We mention, as in Remark 11, that the differences between options (1) and (2) lead to different results regarding monotonicity. In fact, with option (2), the hrm is nonmonotonic with respect to both the addition of cases to the case base, and to addition of information to the focus fact situation, the former of which was shown by van Woerkom et al. (2023b, Proposition 4.18).

### Relation to the RM

We will now show that the hrm is a conservative extension of the rm, in the sense that when the hrm is restricted to hierarchies with only one abstract factor, and case bases which are defined on this factor, it reduces to the rm. To show this, we will construct a translation *f* that maps instances of the rm to instances of the hrm, and prove that this translation respects Definitions 3 and 19 of constraint.

#### Definition 28

A factor hierarchy is *flat* if its set of abstract factors is a singleton.

Any factor partition $F = {{\,\textrm{Pro}\,}}\cup {{\,\textrm{Con}\,}}$ can be mapped to a flat hierarchy $f({{\,\textrm{Pro}\,}}, {{\,\textrm{Con}\,}})$. To do this, we first introduce a new factor $p_\pi$ (so $p_\pi \not \in F$), and then define

$$\begin{aligned} f({{\,\textrm{Pro}\,}}, {{\,\textrm{Con}\,}}) = (F \cup \{p_\pi \}, {{\,\textrm{Pro}\,}}\times \{p_\pi \}, {{\,\textrm{Con}\,}}\times \{p_\pi \}). \end{aligned}$$

It is easily checked that $f({{\,\textrm{Pro}\,}}, {{\,\textrm{Con}\,}})$ satisfies the requirements of Definition 18, and that the set of abstract factors of $f({{\,\textrm{Pro}\,}}, {{\,\textrm{Con}\,}})$ is the singleton $\{p_\pi \}$. Next, we extend *f* to operate on fact situations, cases, and case bases. Given a fact situation *X* for the rm (with respect to the factor partition $({{\,\textrm{Pro}\,}}, {{\,\textrm{Con}\,}})$) we translate it to a fact situation *f*(*X*) for the hrm (with respect to the factor hierarchy $f({{\,\textrm{Pro}\,}}, {{\,\textrm{Con}\,}})$) by defining f(X)(p)=X(p) if $p \in {{\,\textrm{dom}\,}}(X)$, and leaving it undefined otherwise. Similarly, a case (*X*, *s*) can be translated to a case *f*(*X*, *s*) by defining *f*(*X*, *s*)(*p*) for $p \in F \cup \{\pi \}$ by

$$\begin{aligned} f(X, s)(p) = {\left\{ \begin{array}{ll} X(p) & \text {if }p \in {{\,\textrm{dom}\,}}(X), \\ \textbf{t}& \text {if }p = p_\pi \text { and }s = \pi , \\ \textbf{f}& \text {if }p = p_\pi \text { and }s = \delta . \end{array}\right. } \end{aligned}$$

Lastly, given a case base $\mathcal {C}$ we write $f[\mathcal {C}] = \{f(X, s) \mid (X, s) \in \mathcal {C}\}$.

The translation *f* preserves and reflects constraint, in the following sense.

#### Theorem 29

Given a case base $\mathcal {C}$ for a factor partition $F = {{\,\textrm{Pro}\,}}\cup {{\,\textrm{Con}\,}}$ and a focus fact situation *X* we have

$$\begin{aligned}\mathcal {C}, X \vDash \pi \text { iff } f[\mathcal {C}], f(X) \vDash p_\pi \quad \text {and}\quad \mathcal {C}, X \vDash \delta \text { iff } f[\mathcal {C}], f(X) \vDash \lnot p_\pi .\end{aligned}$$

#### Proof

We consider the first equivalence. Spelling out Definition 19 we get

$$\begin{aligned}&f[\mathcal {C}], f(X) \vDash p_\pi \\&\text {iff } \bullet f(X) \vDash p_\pi , \text { or}\\&\quad \bullet p_\pi \in A\text { and there is a case }Y \in f[\mathcal {C}]\text { with }Y \vDash p_\pi \text { and}\\&\qquad \quad \text {-- for all }q \in \textsf{Pro}(p_\pi )\!\!:\text { if }Y \vDash q\text { then }f[\mathcal {C}], f(X) \vDash q,\text { and }\\&\qquad \quad \text {-- for all }q \in \textsf{Con}(p_\pi )\!\!:\text { if }Y \vDash \lnot q\text { then }f[\mathcal {C}], f(X) \vDash \lnot q. \end{aligned}$$

By definition, , and so only the second disjunct in this statement remains. The requirement that $p_\pi \in A = \{p_\pi \}$ holds, and so can be removed. We also know that $\textsf{Pro}(p_\pi ) = {{\,\textrm{Pro}\,}}$ and $\textsf{Con}(p_\pi ) = {{\,\textrm{Con}\,}}$, so these can be substituted. Further filling in the shape of $Y \in f[\mathcal {C}]$ as *f*(*Y*, *s*) for some $(Y, s) \in \mathcal {C}$ we can thus continue with:

$$\begin{aligned}&\text {iff there is a case }(Y, s) \in \mathcal {C}\text { with }f(Y, s) \vDash p_\pi \text { and}\\&\quad \text {-- for all }q \in {{\,\textrm{Pro}\,}}\!\!:\text { if }f(Y, s) \vDash q\text { then }f[\mathcal {C}], f(X) \vDash q,\text { and }\\&\quad \text {-- for all }q \in {{\,\textrm{Con}\,}}\!\!:\text { if }f(Y, s) \vDash \lnot q\text { then }f[\mathcal {C}], f(X) \vDash \lnot q. \end{aligned}$$

By definition of *f*(*Y*, *s*), the requirement that $f(Y, s) \vDash p_\pi$ just reduces to s=π; and, again by definition, f(Y,s)⊨q holds iff Y⊨q. Since the hierarchy is flat, the factors over which the ‘for all’ statements are quantifying are base-level, and so the statement that $f[\mathcal {C}], f(X) \vDash q$ reduces to f(X)⊨q by Lemma 23, which is in turn equivalent to X⊨q by definition of *f*(*X*). We thus continue with:

$$\begin{aligned}&\text {iff there is a case }(Y, \pi ) \in \mathcal {C}\text { such that}\\&\quad \text {-- for all }q \in {{\,\textrm{Pro}\,}}\!\!:\text { if }Y \vDash q\text { then }X \vDash q,\text { and }\\&\quad \text {-- for all }q \in {{\,\textrm{Con}\,}}\!\!:\text { if }Y \vDash \lnot q\text { then }X \vDash \lnot q\\&\text {iff }\mathcal {C}, X \vDash s. \end{aligned}$$

The proof of the second equivalence is very similar to the above, so we omit it. □

## The dimensional result model

In this section we discuss Horty’s (2019) adaptation of the result model to incorporate dimensional information, and extend it to operate on the basis of partial fact situations, in the same way we did for the rm. We start in Sect. 5.1 by adapting our running example from Sect. 3.1 to incorporate dimensional information. We then give Horty’s formalization of dimensions as a knowledge representation in Sect. 5.2, and his accompanying notion of constraint in Sect. 5.3. Then, in Sect. 5.4, we modify the knowledge representation to allow fact situations to be partial, and give a corresponding notion of constraint. We refer to the resulting model as the drm, and go on to show in Sect. 5.5 that it is monotonic in the addition of cases to the case base as well as in the addition of information to the focus fact situation. We conclude in Sect. 5.6 by showing that the drm is a conservative extension of the rm.

### An example of dimensions

A factor can be thought of as an abstract binary property of situations, which either applies to a particular situation or not (regardless of whether this status is known to the decision-maker). A *dimension*, on the other hand, is a property of situations which can take a value from some set of possible values. In this sense, a factor is a special case of a dimension—one which takes a value from a two-element set. Usage of this terminology in the field of ai & law dates back to cato’s predecessor hypo (Ashley 1991).

For an example of dimensions, we return to our running example; consider the following illustration of some dimensions affecting an assessment of recidivism risk:

**Figure:**
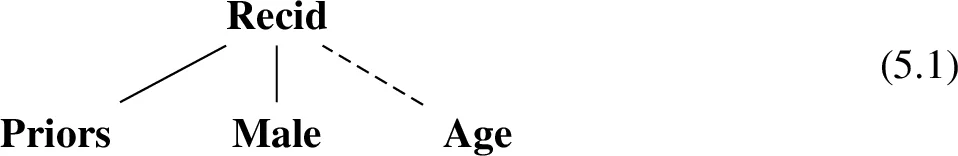

Previously, the ${\textbf {Age}}$ factor represented whether the defendant was over the age of 21. Viewed as a dimension, ${\textbf {Age}}$ can take any value above 0. Similarly, we replace the ${\textbf {Record}}$ factor with a dimension ${\textbf {Priors}}$, indicating the number of previous convictions. The ${\textbf {Male}}$ factor remains as it was, but is now regarded as a binary dimension.

Generally we do not say directly of a dimension whether it favors one of the two outcomes of a case. Instead, we require the dimension to come with a relation expressing the relative preference the values have for the final judgement. Research on recidivism has pointed out that in general older people tend to recidivate less, and so for the ${\textbf {Age}}$ dimension we can say that the value 30 is less indicative than the value 21. In the graph above we have again used solid and dotted links to indicate whether higher values of the dimension are suggestive of high or low risk of recidivism. Dimensions with two values, such as the ${\textbf {Male}}$ dimension, can be considered as factors in the rm and hrm models.

In graph (5.1) above we have again used solid and dotted links to indicate whether higher values of the dimension are suggestive of high or low risk of recidivism. Dimensions with two values, such as the ${\textbf {Male}}$ dimension, can be considered as factors in the rm and hrm models. The polarity of the links chosen here is in line with research on the topic of recidivism risk, see for example the data analysis by Angelino et al. (2018).

### Knowledge representation

We now describe the knowledge representation of the drm as used by Horty (2019). A *dimension* is a nonempty set; we denote dimensions by lower case letters *d*, *e*, *f*, etc. The domain is modeled by a finite set of dimensions *D*. A *fact situation* *X* is a choice function on *D*, i.e. a function $X: D \rightarrow \bigcup D$ such that X(d)∈d for every d∈D.

#### Remark 30

Note that some care should be taken to avoid dimensions “collapsing” in the set-theoretic foundations of the theory. For example, in our running example in the criminal justice domain, we could have two dimensions $\textbf{Priors}_{\text {misd}}$ and $\textbf{Priors}_{\text {fel}}$, which respectively model the number of prior misdemeanor offenses and the number of prior felony offenses. With respect to influence on a high/low judgement of recidivism risk, these would both be modeled as the set of natural numbers ordered by the less-than relation, and therefore would become equal as sets—so $\textbf{Priors}_{\text {misd}} = \textbf{Priors}_{\text {fel}}$. To avoid this we should ensure that they are treated as distinct sets, even if they have the same underlying set of values. This can be achieved in a variety of ways, such as by using tagged sets or indexed sets to distinguish between the two dimensions. For the sake of simplicity we will henceforth leave such distinctions implicit.

Cases are again decided for one of the two sides π or δ, and again we assume that specific values of dimensions have a preference for either of these sides, but this is now modeled by a binary relation on the dimension. More specifically, we assume there is for each dimension d∈D a *preference* relation ⪯ on *d*, which we require to be a partial order. Given values v,w∈d such that v⪯w, we say *w* prefers outcome π relative to *v*, and *v* prefers outcome δ relative to *w*.

### Constraint

The notion of constraint for the drm can now be stated succinctly as follows.

#### Definition 31

The decision of a fact situation *X* is *forced* for π by a case base $\mathcal {C}$, denoted $\mathcal {C}, X \vDash \pi$, if there is a case $(Y, \pi ) \in \mathcal {C}$ with Y(d)⪯X(d) for all d∈D. Similarly, the decision is *forced* for δ, denoted $\mathcal {C}, X \vDash \delta$, if there is $(Y, \delta ) \in \mathcal {C}$ such that X(d)⪯Y(d) for all d∈D.

The factors of the knowledge representation for the rm can be modeled in the drm as two-element dimensions. More specifically, given a factor p∈F for some set of factors *F*, we can construct a dimension $d_p = \{\textbf{f}_p, \textbf{t}_p\}$ which contains exactly two elements corresponding to whether *p* applies or not. If $p \in {{\,\textrm{Pro}\,}}(\pi )$ then we define a relation ≺ on $d_p$ by $\textbf{f}_p \prec \textbf{t}_p$, and if $p \in {{\,\textrm{Pro}\,}}(\delta )$ then we define ≺ by $\textbf{t}_p \prec \textbf{f}_p$. This procedure gives us a method to turn an instance of the rm into an instance of the drm, and the notion of constraint in the drm behaves on these translated instances just as the notion of constraint of the rm behaves on the untranslated instances.

### Partial fact situations

As in the previous sections, we consider a modification of the drm by allowing fact situations to be partial. A partial dimensional fact situation is a partial choice function on the set of dimensions, i.e. a function $X: E \rightarrow \bigcup D$ such that X(d)∈d for every d∈E, where *E* is some subset E⊆D. We denote this domain *E* of *X* by ${{\,\textrm{dom}\,}}(X)$.

Again this raises the question of whether, and if so how, Definition 31 of constraint should be modified to account for this. In Sect. 3.4 we outlined our position regarding constraint induced by partial cases, and it can be applied in the same way to constraint for the drm: if the value of the precedent case in a dimension added strength to the side for which it was decided, then the focus fact situation should have equal-or-greater strength along that dimension for that side.

Consider, for example, a precedent case (Y,π) and a focus fact situation *X*; what conditions need to be met in order for (Y,π) to constrain the decision of *X* to *s*? If *d* is a dimension such that *Y*(*d*) is undefined, then the value of *Y* on this dimension did not add strength to the side of the plaintiff and so no restrictions should be placed on *X*(*d*). If, instead, *d* is a dimension on which *Y*(*d*) is defined then it is difficult to quantify whether *Y*(*d*) added strength for the plaintiff, and so we should require that Y(d)⪯X(d). In particular, if *X*(*d*) is undefined then *X* does not have equal-or-greater strength along this dimension, and so no constraint should be induced. There is, however, one edge case to take into account: when *Y*(*d*) is the least element of *d*—meaning it satisfies Y(d)⪯v for every v∈d—then any value of *X*(*d*) will match or exceed the strength for the side of the plaintiff in that dimension. Therefore, we should allow (Y,π) to induce constraint for *X* in this scenario. This is similar to the situation in the third row of Table 1: if $p \in {{\,\textrm{Con}\,}}$ and Y⊨p, then regardless of whether *p* applies in the focus fact situation *X* or not, it will match the strength of *Y* for the plaintiff for that factor, and so constraint is not excluded.

To capture these considerations in the definition of constraint we introduce the following shorthands:^4^

5.2 $$\begin{aligned} {{\,\textrm{supp}\,}}(Y)&= \{d \in {{\,\textrm{dom}\,}}(Y) \mid Y(d)\text { is not the least element of }d\}, \end{aligned}$$

5.3 $$\begin{aligned} {{\,\textrm{opp}\,}}(Y)&= \{d \in {{\,\textrm{dom}\,}}(Y) \mid Y(d)\text { is not the greatest element of }d\}. \end{aligned}$$

We now define constraint as follows, which (as mentioned earlier) is similar to the approach taken by Prakken (2021, Section 7).

#### Definition 32

The decision of a partial fact situation *X* is *forced* for π by a case base $\mathcal {C}$, denoted $\mathcal {C}, X \vDash \pi$, if there is a partial case $(Y, \pi ) \in \mathcal {C}$ such that for all $d \in {{\,\textrm{supp}\,}}(Y)$: Y(d)⪯X(d). Similarly, the decision is *forced* for δ, denoted $\mathcal {C}, X \vDash \delta$, if there is $(Y, \delta ) \in \mathcal {C}$ such that for all $d \in {{\,\textrm{opp}\,}}(Y)$: X(d)⪯Y(d).

As before, Definitions 31 and 32 coincide for complete fact situations. In what follows we focus on partial fact situations and leave the treatment of complete fact situations implicit.

#### Example 33

We consider an example to demonstrate the behavior of Definition 32, based on the set of dimensions illustrated in (5.1). These dimensions are given by the sets:

$$\begin{aligned} {\textbf {Priors}}&= \{0, 1, 2, \dots \}, \\ {\textbf {Male}}&= \{0, 1\}, \\ {\textbf {Age}}&= \{18, 19, 20, \dots \}. \end{aligned}$$

The associated orders are the usual less-than relation ≤ on the natural numbers for the **Priors** and **Male** dimensions, while for **Age** we take its inverse ≥. Suppose that upon these dimensions a judgement is made about a defendant being at high (π) or low (δ) risk of recidivism. Let (Y,π) be a case with a fact situation *Y* given by $Y({\textbf {Priors}}) = 10$, $Y({\textbf {Male}}) = 0$, and $Y({\textbf {Age}}) = 20$. What constraint does the case base $\{(Y, \pi )\}$ induce on a focus fact situation *X*? Applying Definition 32 we find:

$$\begin{aligned}&\{(Y, \pi )\}, X \vDash \pi \\&\text {iff for all }d \in {{\,\textrm{supp}\,}}(Y): Y(d) \preceq X(d) \\&\text {iff }Y({\textbf {Priors}}) \preceq X({\textbf {Priors}})\text { and }Y({\textbf {Age}}) \preceq X({\textbf {Age}}) \\&\text {iff }10 \le X({\textbf {Priors}})\text { and }20 \ge X({\textbf {Age}}). \end{aligned}$$

In other words, any defendant which is 20 or younger and has 10 or more prior offenses is constrained to be judged at a high risk of recidivism by the earlier decision (Y,π). Note, in particular, that *X* may be undefined on the **Male** dimension, since any value that it would assign would be above 0 as this is the least element of the dimension.

### Monotonicity

Like the factor-based models, the drm is monotonic in the case base.

#### Proposition 34

If $\mathcal {C}\subseteq \mathcal {D}$ then $\mathcal {C}, X \vDash s$ implies $\mathcal {D}, X \vDash s$ for $s \in \{\pi , \delta \}$.

#### Proof

Suppose s=π, and let *X* be a fact situation such that $\mathcal {C}, X \vDash \pi$, then this means there is a case $(Y, \pi ) \in \mathcal {C}$ satisfying the required conditions of Definitions 32, and so since $\mathcal {C}\subseteq \mathcal {D}$ we get $(Y, \pi ) \in \mathcal {D}$ and $\mathcal {D}, X \vDash \pi$ as desired. The case for s=δ is similar. □

Fact situations are again modelled by functions, so we can define the addition of information to fact situations in terms of function extension in the sense of Definition 8.

#### Proposition 35

If X⊆Y then $\mathcal {C}, X \vDash s$ implies $\mathcal {C}, Y \vDash s$ for $s \in \{\pi , \delta \}$.

#### Proof

Suppose s=π. Since $\mathcal {C}, X \vDash \pi$ there is $(Z, \pi ) \in \mathcal {C}$ such that Z(d)⪯X(d)=Y(d) for all $d \in {{\,\textrm{supp}\,}}(Z)$, and so $\mathcal {C}, Y \vDash \pi$ as desired. The case for s=δ is similar. □

### Relation to the RM

We will now show that the drm is a conservative extension of the rm, in the sense that when the drm is restricted to binary dimensions with linear orders, it reduces to the rm. To show this, we construct a translation *f* that maps instances of the rm to instances of the drm, and prove that this translation respects Definitions 3 and 32 of constraint.

#### Definition 36

A set of dimensions *D* is *binary* if each d∈D has cardinality 2 and is ordered linearly.

Let $F = {{\,\textrm{Pro}\,}}\cup {{\,\textrm{Con}\,}}$ be a factor partition, for each p∈F we define a binary dimension $d_p = \{\textbf{t}_p, \textbf{f}_p\}$. The associated order for $d_p$ is the reflexive closure of $\textbf{f}_p \prec \textbf{t}_p$ if $p \in {{\,\textrm{Pro}\,}}$, and that of $\textbf{t}_p \prec \textbf{f}_p$ if $p \in {{\,\textrm{Con}\,}}$. We write $f[F] = \{d_p \mid p \in F\}$ for the set of dimensions corresponding to the factor partition *F*. Next, we extend *f* to operate on fact situations, cases, and case bases. Given a fact situation *X* for the rm (with respect to the factor partition *F*) we translate it to a fact situation *f*(*X*) for the drm (with respect to the set of dimensions *f*[*F*]) by defining *f*(*X*) on $d_p$ for $p \in {{\,\textrm{dom}\,}}(X)$:

$$\begin{aligned} f(X)(d_p) = {\left\{ \begin{array}{ll} \textbf{t}_p & \text {if }X \vDash p, \\ \textbf{f}_p & \text {if }X \vDash \lnot p. \end{array}\right. } \end{aligned}$$

Similarly, a case (*X*, *s*) is translated to a case (*f*(*X*), *s*), and given a case base $\mathcal {C}$ for *F* we let $f[\mathcal {C}]$ denote $\{(f(X), s) \mid (X, s) \in \mathcal {C}\}$.

The translation *f* preserves and reflects constraint, in the following sense.

#### Theorem 37

Given a case base $\mathcal {C}$ for a factor partition $F = {{\,\textrm{Pro}\,}}\cup {{\,\textrm{Con}\,}}$ and a focus fact situation *X* we have

$$\begin{aligned} \mathcal {C}, X \vDash \pi \text { iff } f[\mathcal {C}], f(X) \vDash \pi \quad \text {and}\quad \mathcal {C}, X \vDash \delta \text { iff } f[\mathcal {C}], f(X) \vDash \delta . \end{aligned}$$

#### Proof

We consider the first equivalence. Spelling out Definition 32 we get

$$\begin{aligned}&f[\mathcal {C}], f(X) \vDash \pi \\&\text {iff there is }(Y, \pi ) \in f[\mathcal {C}]\text { such that for all }d_p \in {{\,\textrm{supp}\,}}(Y)\!\!: Y(d_p) \preceq f(X)(d_p) \\&\text {iff there is }(Y, \pi ) \in \mathcal {C}\text { such that for all }d_p \in {{\,\textrm{supp}\,}}(f(Y))\!\!: f(Y)(d_p) \preceq f(X)(d_p) \\&\text {iff there is }(Y, \pi ) \in \mathcal {C}\text { such that for all }p \in {{\,\textrm{dom}\,}}(Y)\!\!: \\&\qquad \text { if }f(Y)(d_p)\text { is not the least element of }d_p\text { then }f(Y)(d_p) \preceq f(X)(d_p). \end{aligned}$$

Now either $p \in {{\,\textrm{Pro}\,}}$ or $p \in {{\,\textrm{Con}\,}}$. In the former case, $d_p$ is ordered by $\textbf{f}_p \prec \textbf{t}_p$ and so the least element of $d_p$ is $\textbf{f}_p$. The latter case is dual. We can thus continue with:

$$\begin{aligned}&\text {iff there is }(Y, \pi ) \in \mathcal {C}\text { such that:} \\&\quad \bullet \text { for all }p \in {{\,\textrm{dom}\,}}(Y) \cap {{\,\textrm{Pro}\,}}\!\!:\text { if }f(Y)(d_p) \not = \textbf{f}_p\text { then }f(Y)(d_p) \preceq f(X)(d_p),\text { and} \\&\quad \bullet \text { for all }p \in {{\,\textrm{dom}\,}}(Y) \cap {{\,\textrm{Con}\,}}\!\!:\text { if }f(Y)(d_p) \not = \textbf{t}_p\text { then }f(Y)(d_p) \preceq f(X)(d_p). \end{aligned}$$

If $p \in {{\,\textrm{dom}\,}}(Y) \cap {{\,\textrm{Pro}\,}}$ then *f*(*Y*) is defined on $d_p$ and so $f(Y)(d_p) \not = \textbf{f}_p$ implies $f(Y)(d_p) = \textbf{t}_p$, and furthermore $f(Y)(d_p) = \textbf{t}_p \preceq f(X)(d_p)$ reduces to $f(X)(d_p) = \textbf{t}_p$. Applying similar reasoning to the case for $p \in {{\,\textrm{dom}\,}}(Y) \cap {{\,\textrm{Con}\,}}$ we obtain:

$$\begin{aligned}&\text {iff there is }(Y, \pi ) \in \mathcal {C}\text { such that:} \\&\quad \bullet \text { for all }p \in {{\,\textrm{dom}\,}}(Y) \cap {{\,\textrm{Pro}\,}}\!\!:\text { if }f(Y)(d_p) = \textbf{t}_p\text { then }f(X)(d_p) = \textbf{t}_p,\text { and} \\&\quad \bullet \text { for all }p \in {{\,\textrm{dom}\,}}(Y) \cap {{\,\textrm{Con}\,}}\!\!:\text { if }f(Y)(d_p) = \textbf{f}_p\text { then }f(X)(d_p) = \textbf{f}_p \\&\text {iff there is }Y, \pi ) \in \mathcal {C}\text { such that:} \\&\quad \bullet \text { for all }p \in {{\,\textrm{dom}\,}}(Y) \cap {{\,\textrm{Pro}\,}}\!\!:\text { if }Y(p) = \textbf{t}\text { then }X(p) = \textbf{t},\text { and} \\&\quad \bullet \text { for all }p \in {{\,\textrm{dom}\,}}(Y) \cap {{\,\textrm{Con}\,}}\!\!:\text { if }Y(p) = \textbf{f}\text { then }X(p) = \textbf{f}. \end{aligned}$$

Intersecting with ${{\,\textrm{dom}\,}}(Y)$ is redundant because for undefined values *Y*(*p*) the implication over which is being quantified holds trivially. Furthermore, $Y(p) = \textbf{t}$ just means the same as Y⊨p, and likewise for $Y(p) = \textbf{f}$ and $Y \vDash \lnot p$, so we have:

$$\begin{aligned}&\text {iff there is }(Y, \pi ) \in \mathcal {C}\text { such that:} \\&\quad \bullet \text {for all }p \in {{\,\textrm{Pro}\,}}\!\!:\text { if }Y \vDash p\text { then }X \vDash p,\text { and} \\&\quad \bullet \text { for all }p \in {{\,\textrm{Con}\,}}\!\!:\text { if }Y \vDash \lnot p\text { then }X \vDash \lnot p \\&\text {iff }\mathcal {C}, X \vDash \pi . \end{aligned}$$

The derivation of $\mathcal {C}, X \vDash \delta$ iff $f[\mathcal {C}], f(X) \vDash \delta$ is very similar, so we omit it. □

## The dimensional hierarchical result model

The goal of this section is to unify the knowledge representations of the previous sections, as well as their associated notions of constraint, by considering a set of dimensions which has hierarchical structure—we call this a *dimension hierarchy*. We start in Sect. 6.1 by extending the running example from Sect. 3.1 to incorporate both dimensional and hierarchical information. The notion of dimension hierarchy is then formalized as a knowledge representation in Sect. 6.2, and its associated notion of constraint is given in Sect. 6.3. We refer to the resulting model as the dhrm  and the rest of the sections are dedicated to investigating some of its formal properties. In particular, in Sect. 6.4, we show that the dhrm is monotonic in both the addition of cases to the case base and in addition to information to a focus fact situation. Then, in Sects. 6.5 and 6.6 we show that the dhrm is a conservative extension of both the hrm and the drm, respectively.

### An example of a dimension hierarchy

A major difference between the drm and the dhrm is that dimensions can now be subordinate to multiple, more abstract factors. This raises the question whether the dimension preference orders ⪯ should be specified per dimension or per link in the hierarchy. Additionally, dimensions are now subordinate to other dimensions, rather than to a (binary) case outcome. What does it mean for a dimension to influence a more abstract dimension? We will illustrate these differences through some examples to build intuition, before continuing to the formal definitions.

Consider the following modification of our running example of (4.1):

**Figure:**
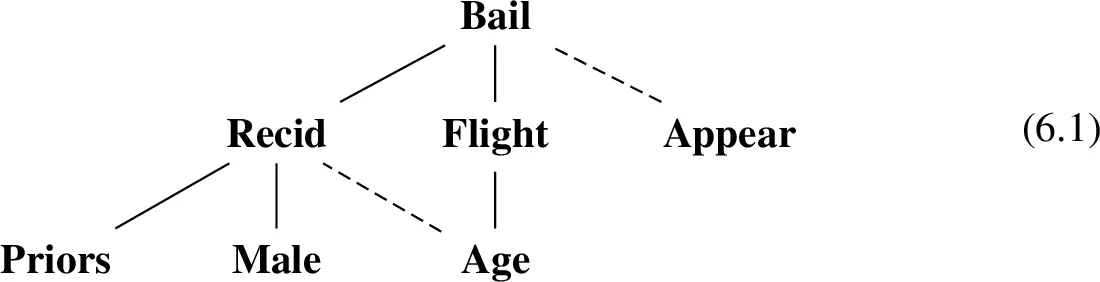

In the setting of a dimension hierarchy we can consider recidivism risk as a dimension, for instance as a score ranging from 1 to 10. The compas system also outputs a score from 1 to 10 indicating severity of the risk, so this is a more realistic example than those in the previous sections. Additionally, ${\textbf {Bail}}$ can now be considered a dimension, specifying the amount of bail in, say, usd. Note that denial of bail can still be modeled as an ‘infinite’ amount of bail. ${\textbf {Appear}}$, too, can be considered a dimension, indicating the relative frequency of past trial appearances by the defendant.

**Table 2:**
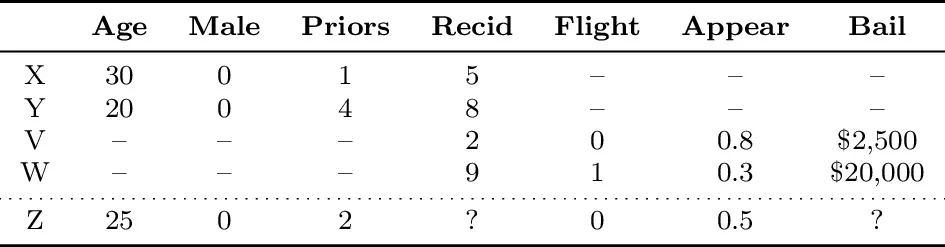
Five example fact situations *V*, *W*, *X*, *Y*, and *Z* for the bail domain

To begin building some intuition for an appropriate notion of constraint in this setting we illustrate a difference with the drm, which is that dimensions now affect other dimensions instead of the case outcome directly. To this end, we consider the subgraph of (6.1) consisting of just the dimensions ${\textbf {Recid}}$, ${\textbf {Priors}}$, ${\textbf {Male}}$, and ${\textbf {Age}}$, and the fact situations *X*, *Y*, and *Z*, listed in Table 2. The situation *Z* concerns a 25-year-old female with 2 prior offenses. What recidivism risk score may be consistently assigned to *Z*, given the previous judgments that a 30-year-old female with 1 prior offense received score 5 (situation *X*), and that a 20-year-old male with 4 prior offenses received score 8 (situation *Y*)? Comparing the situation *Z* to *X* we see that *Z* is dimension-wise equal or more indicative of recidivism risk than *X*: *Z* is younger, both *Z* and *X* are female, and *Z* has more prior offenses. Since *X* received a recidivism risk score of 5, it seems sensible to require that *Z* would get *at least* a score of 5, but possibly higher since *Z* is indicative of higher risk on some dimensions. This exemplifies one of the key differences between the dhrm and the previous models—decisions are not forced exactly, but constrained to lie within an interval. Comparing *Z* to the situation *Y* we get the opposite picture; *Y* has received a risk score of 8, but *Z* is dimension-wise equal or less indicative of recidivism risk than *Y*. Therefore, we expect *Z* to receive a score of *at most* 8. In sum, $\{X, Y\}$ should produce the constraint that $5 \preceq Z({\textbf {Recid}}) \preceq 8$.

We now turn our attention to the full hierarchy, depicted by graph (6.1), involving a downstream judgement of bail amount. In such a scenario, we can apply a recursive notion of constraint as in the hrm. Consider, again, the fact situations listed in Table 2. We have seen that *X* and *Y* bind the recidivism score of *Z* to the integer range [5, 8]. In addition, we now have two situations *V* and *W* for which a bail amount was determined on the basis of their recidivism risk assessment, risk of flight, and relative frequency of previous trial appearances. We omit the information that led to the recidivism score assignment for these fact situations. This is a general feature of the hierarchical models—once an abstract dimension has been given a value it can be used for downstream reasoning regardless of the reason behind the value’s assignment. Defendant *V* was granted a bail amount of $$\$2{,}500$$, on the basis of a recidivism risk score of 2, a perceived low risk of flight, and an 80% appearance rate at previous trials. Defendant *Z* has a lower appearance rate at previous trials and is similarly perceived as unlikely to flee, but is not yet assigned a definitive recidivism risk score. However, since we know that *Z* should receive a risk score of at least 5 it will in any case be higher than *V*’s score of 2. Therefore, we would ultimately expect *Z* to receive a bail amount which is equal or greater than that of *V*—so $$\$2{,}500 \preceq Z({\textbf {Bail}})$$. Similarly, we can deduce from the case *W* that, since *Z* should receive a risk score of at most 8, the amount of bail for *Z* should not exceed $20,000—so $$Z({\textbf {Bail}}) \preceq \$20{,}000$$. In sum, the case base $$\{V, W, X, Y\}$$ should produce the constraints $$\$2{,}500 \preceq Z({\textbf {Bail}}) \preceq \$20{,}000$$.

This use of recursion is useful, because it allows the use of the forcing relation despite some dimensions not having been assigned an exact value. Consider, for instance, the decision support system implemented by the Dutch national police force (Odekerken and Bex 2020). It is argued by Odekerken et al. (2023) that determining the values of dimensions for a specific case can be costly, and the aforementioned use of recursion can alleviate this need for abstract factors.

### Knowledge representation

We now adapt Definition 18 of a factor hierarchy to that of a dimension hierarchy.

#### Definition 38

A *dimension hierarchy* is a triple $(D, \textsf{Pro}, \textsf{Con})$, where *D* is a finite set of dimensions, and $\textsf{Pro}$ and $\textsf{Con}$ are relations on *D* such that $\textsf{Pro}\cap \textsf{Con}= \emptyset$, and such that the transitive closure of $\textsf{H}= \textsf{Pro}\cup \textsf{Con}$ is irreflexive.

We maintain the same terminology as in the hrm for factors with respect to their position in the hierarchy—a dimension is *base-level* if it is $\textsf{H}$-minimal, and the set of base level dimensions is denoted by *B*; if a dimension is not base-level then it is *abstract*, and we write *A* for the set of abstract dimensions. A *fact situation* *X* is a choice function on a subset of *D*; we denote its domain by ${{\,\textrm{dom}\,}}(X)$. Lastly, we assume each dimension d∈D is assigned a partial order ⪯ on *d*.

The way we define a dimension hierarchy in this section is with a fixed order for every node of the hierarchy; outgoing positive links represent a positive correlation with respect to this order, and negative links represent a negative correlation. The reader may wonder about the situation when a dimension has an influence on multiple more abstract dimension—but with respect to different orders. Consider, for example, an **Education** dimension, with three possible values: none, high school, or higher education. With respect to influence on risk of recidivism, we may order these values by $\texttt {higher}\ \texttt {education}\prec \texttt {high}\ \texttt {school}\prec \texttt {none}$; but **Education** may also influence other dimensions with neither this nor its inverse order. For example, a judge may take into account the defendant’s societal ties when determining flight risk. Having had a form of education could be taken as reducing flight risk, without differentiating specifically between a high school diploma and higher education. So, with respect to flight risk we could use an ordering with $\texttt {none}\prec \texttt {high}\ \texttt {school}$ and $\texttt {none}\prec \texttt {higher}\ \texttt {education}$, but with high school and higher education mutually incomparable.

One way to deal with this would be to associate orders with links, rather than with dimensions directly. In the previous example, this would mean that the **Education** dimension has one outgoing link with respect to the linear order $\texttt {higher}\ \texttt {education}\prec \texttt {high}\ \texttt {school}\prec \texttt {none}$, and one with respect to the order in which high school  and higher education  are incomparable. The difficulty with this approach is that, if a dimension does not have a single order with which it is associated, it becomes cumbersome to specify what it means for two dimensions to be positively correlated. For example, we would like to interpret a positive link of the form d→e in a hierarchy to mean that “a higher value for *d* tends to imply a higher value for *e*”, but the meaning of this statement would be unclear in a scenario where there are multiple orderings associated to the dimension *e*. To deal with that, each link of the form d→e in the hierarchy would need to specify a dimension order on *d* and on *e*, and thus incur an additional bookkeeping cost. The resulting notion of constraint would seem to become more complex than the one we will shortly propose in Definition 39.

We thus opt for a simpler solution: fix one order for every dimension, and let the dimension influence and be influenced only according to this fixed order. To deal with cases where a dimension has an influence with respect to multiple different orders, such as the aforementioned **Education** dimension, we require the dimension to be added multiple times to the hierarchy, with one instance for each of the orders with which it has an influence on other dimensions. We think the price of the redundancy in this representation is compensated by a simpler definition of constraint.

### Constraint

As in the hrm we define $\textsf{Pro}(d) = \{e \in D \mid \textsf{Pro}(e, d)\}$. Using this, together with the definitions of support and opposition of (5.2) and (5.3), we now define by mutual recursion two relations $\mathcal {C}\vDash v \preceq X(d)$ and $\mathcal {C}\vDash X(d) \preceq v$, indicating constraint in the form of lower and upper bounds for *X*(*d*) in a focus fact situation *X*.

#### Definition 39

Given a case base $\mathcal {C}$ and a value *v* in some dimension *d*, a fact situation *X* is *lower bounded* by *v* and $\mathcal {C}$, written $\mathcal {C}\vDash v \preceq X(d)$, if and only if either

- v⪯X(d), or
- d∈A and there is $Y \in \mathcal {C}$ such that v⪯Y(d) and
  - for all $e \in \textsf{Pro}(d) \cap {{\,\textrm{supp}\,}}(Y)$: $\mathcal {C}\vDash Y(e) \preceq X(e)$, and
  - for all $$e \in \textsf{Con}(d) \cap {{\,\textrm{opp}\,}}(Y)$$: $$\mathcal {C}\vDash X(e) \preceq Y(e)$$.

Similarly, the *upper bound* by *v*, written $\mathcal {C}\vDash X(d) \preceq v$, is defined to hold iff:

- X(d)⪯v, or
- d∈A and there is $Y \in \mathcal {C}$ such that Y(d)⪯v and
  - for all $e \in \textsf{Pro}(d) \cap {{\,\textrm{opp}\,}}(Y)$: $\mathcal {C}\vDash X(e) \preceq Y(e)$, and
  - for all $$e \in \textsf{Con}(d) \cap {{\,\textrm{supp}\,}}(Y)$$: $$\mathcal {C}\vDash Y(e) \preceq X(e)$$.

The idea behind the recursive clause is that there is a precedent *Y* which, by the a fortiori principle, forces *X*(*d*) to take a value which is at least *Y*(*d*), and therefore v⪯X(d) follows by transitivity from $v \preceq Y(d) \preceq X(d)$. Note that, by definition, $\mathcal {C}\vDash v \preceq X(d)$ is always false when *X* is undefined on *d*.

We can also define strict versions of the forcing relations.

#### Definition 40

A fact situation *X* is *strictly lower bounded* by a case base $\mathcal {C}$ to some value v∈d, denoted $\mathcal {C}\vDash v \prec X(d)$, if there is v≺w such that $\mathcal {C}\vDash w \preceq X(d)$; the *strict upper bound* $\mathcal {C}\vDash X(d) \prec v$ is defined similarly.

Analogous to Lemmas 22 and 23 we have some simple consequences of Definition 39.

#### Lemma 41

v⪯X(d) implies $\mathcal {C}\vDash v \preceq X(d)$, and X(d)⪯v implies $\mathcal {C}\vDash X(d) \preceq v$.

#### Lemma 42

If d∈B then $\mathcal {C}\vDash v \preceq X(d)$ implies v⪯X(d) and $\mathcal {C}\vDash X(d) \preceq v$ implies X(d)⪯v.

#### Example 43

To verify Definition 39 correctly captures the intuition of the example in Sect. 6.1 we now consider a dimension hierarchy $(D, \textsf{Pro}, \textsf{Con})$ as depicted in (6.1), so

$$\begin{aligned} D&= \{{\textbf {Priors}}, {\textbf {Male}}, {\textbf {Age}}, {\textbf {Recid}}, {\textbf {Flight}}, {\textbf {Appear}}, {\textbf {Bail}}\}, \\ {\textbf {Priors}}&= \{0, 1, 2, \dots \}, \\ {\textbf {Male}}&= \{0, 1\}, \\ {\textbf {Age}}&= \{18, 19, 20, \dots \}, \\ {\textbf {Recid}}&= \{1, 2, \dots , 9, 10\}, \\ {\textbf {Flight}}&= \{0, 1\}, \\ {\textbf {Appear}}&= [0, 1], \\ {\textbf {Bail}}&= \{\$x \mid x \in \{0, 1, 2, \dots \}\}, \\ \textsf{Pro}&= \{({\textbf {Priors}}, {\textbf {Recid}}), ({\textbf {Male}}, {\textbf {Recid}}), ({\textbf {Age}}, {\textbf {Flight}}), ({\textbf {Recid}}, {\textbf {Bail}}), \\&\qquad \, ({\textbf {Flight}}, {\textbf {Bail}})\},\\ \textsf{Con}&= \{({\textbf {Age}}, {\textbf {Recid}}), ({\textbf {Appear}}, {\textbf {Bail}})\}. \end{aligned}$$

The dimension orders are all just given by the usual less-than order ≤. We let *W*, *Y*, and *Z* be as listed in Table 2. The question is now whether $\{W, Y\} \vDash Z({\textbf {Bail}}) \preceq \$20{,}000$. To check this, we first verify that $\{W, Y\} \vDash Z({\textbf {Recid}}) \preceq 9$:

$$\begin{aligned}&\{W, Y\} \vDash Z({\textbf {Recid}}) \preceq 9 \\&\text {if there is }T \in \{W, Y\}\text { such that }T({\textbf {Recid}}) \preceq 9\text { and} \\&\quad \bullet \text { for all }d \in \textsf{Pro}({\textbf {Recid}}) \cap {{\,\textrm{opp}\,}}(T)\!\!: \{W, Y\} \vDash Z(d) \preceq T(d), \text { and} \\&\quad \bullet \text { for all }d \in \textsf{Con}({\textbf {Recid}}) \cap {{\,\textrm{supp}\,}}(T)\!\!: \{W, Y\} \vDash T(d) \preceq Z(d). \\ \end{aligned}$$

We may now substitute T=Y as $Y({\textbf {Recid}}) = 8 \preceq 9$:

$$\begin{aligned} \text {if }&\bullet \text { for all }d \in \textsf{Pro}({\textbf {Recid}}) \cap {{\,\textrm{opp}\,}}(Y)\!\!: \{W, Y\} \vDash Z(d) \preceq Y(d), \text { and} \\&\bullet \text { for all }d \in \textsf{Con}({\textbf {Recid}}) \cap {{\,\textrm{supp}\,}}(Y)\!\!: \{W, Y\} \vDash Y(d) \preceq Z(d). \end{aligned}$$

Note that $\textsf{Pro}({\textbf {Recid}}) \cap {{\,\textrm{opp}\,}}(Y) = \{{\textbf {Priors}}\}$ (${\textbf {Male}}\not \in {{\,\textrm{opp}\,}}(Y)$ as $Y({\textbf {Male}}) = 1$ is its greatest element) and $\textsf{Con}({\textbf {Recid}}) \cap {{\,\textrm{supp}\,}}(Y) = \{{\textbf {Age}}\}$, so this evaluates to

$$\begin{aligned}&\text {if } \{W, Y\} \vDash Z({\textbf {Priors}}) \preceq Y({\textbf {Priors}}) \text { and } \{W, Y\} \vDash Y({\textbf {Age}}) \preceq Z({\textbf {Age}}). \end{aligned}$$

All dimensions subordinate to ${\textbf {Recid}}$ are base-level so this simplifies by Lemma 42 to:

$$\begin{aligned}&\text {if } Z({\textbf {Priors}})\le 4 \text { and } 20 \le Z({\textbf {Age}}). \end{aligned}$$

Indeed, defendant *Z* of Table 2 satisfies these conditions and so $\{W, Y\} \vDash Z({\textbf {Recid}}) \preceq 9$. Next, we proceed in the same fashion to confirm that $\{W, Y\} \vDash Z({\textbf {Bail}}) \preceq \$20{,}000$:

$$\begin{aligned}&\{W, Y\} \vDash Z({\textbf {Bail}}) \preceq \ 20{,}000 \\&\text {if there is }T \in \{W, Y\}\text { such that }T({\textbf {Bail}}) \preceq \ 20{,}000 \text { and} \\&\bullet \text { for all }d \in \textsf{Pro}({\textbf {Bail}}) \cap {{\,\textrm{opp}\,}}(T)\!\!: \{W, Y\} \vDash Z(d) \preceq T(d), \text { and} \\&\bullet \text { for all }d \in \textsf{Con}({\textbf {Bail}}) \cap {{\,\textrm{supp}\,}}(T)\!\!: \{W, Y\} \vDash T(d) \preceq Z(d) \\&\text {if } \bullet \text { for all }d \in \textsf{Pro}({\textbf {Bail}}) \cap {{\,\textrm{opp}\,}}(Y)\!\!: \{W, Y\} \vDash Z(d) \preceq Y(d), \text { and} \\&\bullet \text { for all }d \in \textsf{Con}({\textbf {Bail}}) \cap {{\,\textrm{supp}\,}}(Y)\!\!: \{W, Y\} \vDash Y(d) \preceq Z(d) \\&\text {if } \{W, Y\} \vDash Z({\textbf {Recid}}) \preceq 9, \text { and } \{W, Y\} \vDash 0.3 \preceq Z({\textbf {Appear}}) \\&\text {if } \{W, Y\} \vDash Z({\textbf {Recid}}) \preceq 9 \text { and } 0.3 \preceq Z({\textbf {Appear}}). \end{aligned}$$

We have established that $\{W, Y\} \vDash Z({\textbf {Recid}}) \preceq 9$, and so as $0.3 \preceq 0.5 = Z({\textbf {Appear}})$ we indeed have $\{W, Y\} \vDash Z({\textbf {Bail}}) \preceq \$20{,}000$ as desired.

#### Remark 44

A dimension hierarchy, like a factor hierarchy, can contain positive and negative links. It is worth noting that the polarity of these links can be switched by reversing the dimension order associated with the subordinate dimension. Consider, as an example, the negative link between **Age** and **Recid** in (6.1). The order associated with **Age** is the usual order ≤ on natural numbers, and so, since older people tend to recidivate less, the **Age** dimension has a negative link to the **Recid** dimension. However, if we choose to pair **Age** with the ≥ order on the natural numbers, then this same relation between age and recidivism would be represented by a positive link. Of course, changing the order of **Age** in this hierarchy would mean that the polarity of the link from **Age** to **Flight** would have to be flipped as well.

### Monotonicity

Like the other models, the dhrm is monotonic in both the addition of new cases and in the addition of information to fact situations.

#### Proposition 45

Let $\mathcal {C}\subseteq \mathcal {D}$ be case bases, *d* a dimension, v∈d a value, and be case bases; then $\mathcal {C}\vDash v \preceq X(d)$ implies $\mathcal {D}\vDash v \preceq X(d)$ and $\mathcal {C}\vDash X(d) \preceq v$ implies $\mathcal {D}\vDash X(d) \preceq v$.

#### Proof

The proof (just as that of Proposition 25) proceeds by induction on the position of *d* in the hierarchy $\textsf{H}$. The base case, when d∈B, follows from Lemmas 41 and 42. When d∈A the induction hypothesis is that for all $e \in \textsf{Pro}(d) \cup \textsf{Con}(d)$ and any value w∈e: $\mathcal {C}\vDash w \preceq X(e)$ implies $\mathcal {D}\vDash w \preceq X(e)$ and $\mathcal {C}\vDash X(e) \preceq w$ implies $\mathcal {D}\vDash X(e) \preceq w$. Now, assume $\mathcal {C}\vDash v \preceq X(d)$. If this is due to v⪯X(d) then $\mathcal {D}\vDash v \preceq X(d)$ follows immediately. So, suppose that it is due to $Y \in \mathcal {C}$ with v⪯Y(d) with

$$\begin{aligned}&-\text { for all }e \in \textsf{Pro}(d) \cap {{\,\textrm{supp}\,}}(Y)\!\!: \mathcal {C}\vDash Y(e) \preceq X(e),\text { and } \\&-\text { for all }e \in \textsf{Con}(d) \cap {{\,\textrm{opp}\,}}(Y)\!\!: \mathcal {C}\vDash X(e) \preceq Y(e). \end{aligned}$$

Applying the induction hypothesis we can conclude that

$$\begin{aligned}&\text {-- for all }e \in \textsf{Pro}(d) \cap {{\,\textrm{supp}\,}}(Y)\!\!: \mathcal {D}\vDash Y(e) \preceq X(e),\text { and } \\&\text {-- for all }e \in \textsf{Con}(d) \cap {{\,\textrm{opp}\,}}(Y)\!\!: \mathcal {D}\vDash X(e) \preceq Y(e). \end{aligned}$$

which is to say that $\mathcal {D}\vDash v \preceq X(d)$. By the same reasoning we can show $\mathcal {C}\vDash X(d) \preceq v$ implies $\mathcal {D}\vDash X(d) \preceq v$, which completes the induction case and thus the proof. □

#### Proposition 46

If X⊆Y then $\mathcal {C}\vDash v \preceq X(d)$ implies $\mathcal {C}\vDash v \preceq Y(d)$, and similarly $\mathcal {C}\vDash X(d) \preceq v$ implies $\mathcal {C}\vDash Y(d) \preceq v$.

#### Proof

We proceed by induction on *d*. In the base case we have by Lemmas 41 and 42 that if $\mathcal {C}\vDash v \preceq X(d)$ then v⪯X(d)=Y(d), and so $\mathcal {C}\vDash v \preceq Y(d)$; and similarly for $\mathcal {C}\vDash X(d) \preceq v$. The induction case proceeds just as in the proof of Proposition 45. □

### Relation to the HRM

We now show that the dhrm is a conservative extension of the hrm, in the sense that when the dhrm is restricted to dimension hierarchies with only binary dimensions, it reduces to the hrm. To show this, we construct a translation *f* that maps instances of the hrm to instances of the dhrm, and prove that this translation respects Definitions 19 and 39 of constraint. Of course, the dhrm constrains fact situations to take values within intervals, rather than forcing them to take specific values, but forcing specific values is easily expressed, for instance by defining $\mathcal {C}\vDash X(d) = v$ as the conjunction of $\mathcal {C}\vDash v \preceq X(d)$ and $\mathcal {C}\vDash X(d) \preceq v$. If *v* is a maximal element of a dimension *d*, meaning there is no *w* in *d* satisfying v≺w, then it suffices to require that $\mathcal {C}\vDash v \preceq X(d)$. This is how we encode hrm-style forcing in the dhrm—by defining a binary dimension $d_p = \{\textbf{f}_p, \textbf{t}_p\}$ with $\textbf{f}_p \prec \textbf{t}_p$ (as in the translation in Theorem 37), and requiring $\mathcal {C}\vDash \textbf{t}_p \preceq X(d_p)$, which amounts to the requirement that $X(d_p)$ takes the value $\textbf{t}_p$.

#### Definition 47

A dimension hierarchy $(D, \textsf{Pro}, \textsf{Con})$ is *binary* if each d∈D has cardinality 2 and is ordered linearly.

Let $(F, \textsf{Con}, \textsf{Pro})$ be a factor hierarchy; for any p∈F we define a binary dimension $d_p = \{\textbf{f}_p, \textbf{t}_p\}$, linearly ordered by the reflexive closure of $\textbf{f}_p \prec \textbf{t}_p$. We now translate $(F, \textsf{Con}, \textsf{Pro})$ to a binary dimension hierarchy $f(F, \textsf{Con}, \textsf{Pro})$ by

$$\begin{aligned}f(F, \textsf{Con}, \textsf{Pro}) = (D, \textsf{Con}', \textsf{Pro}'),\end{aligned}$$

where $D = \{d_p \mid p \in F\}$, $\textsf{Pro}'(d_p, d_q)$ iff $\textsf{Pro}(p, q)$, and similarly $\textsf{Con}'(d_p, d_q)$ iff $\textsf{Con}(p, q)$; so $\textsf{Pro}'(d_p) = \{d_q \in D \mid q \in \textsf{Pro}(p)\}$, and similarly $\textsf{Con}'(d_p) = \{d_q \in D \mid q \in \textsf{Con}(p)\}$. Next, we extend *f* to operate on fact situations and case bases. Given a fact situation *X* for the hrm (with respect to the factor hierarchy $(F, \textsf{Pro}, \textsf{Con})$) we translate it to a fact situation *f*(*X*) for the dhrm (with respect to the dimension hierarchy $f(F, \textsf{Pro}, \textsf{Con})$):

$$\begin{aligned} f(X)(d_p) = {\left\{ \begin{array}{ll} \textbf{t}_p & \text {if }X \vDash p, \\ \textbf{f}_p & \text {if }X \vDash \lnot p, \\ \text {undefined} & \text {otherwise}. \end{array}\right. } \end{aligned}$$

As before, we write $f[\mathcal {C}] = \{f(X) \mid X \in \mathcal {C}\}$ for a case base $\mathcal {C}$.

The translation *f* preserves and reflects constraint, in the following sense.

#### Theorem 48

Given a case base $\mathcal {C}$ for a factor hierarchy $(F, \textsf{Pro}, \textsf{Con})$, a focus fact situation *X*, and a factor p∈F, we have

$$\begin{aligned} \mathcal {C}, X \vDash p \text { iff } f[\mathcal {C}] \vDash \textbf{t}_p \preceq f(X)(d_p) \quad \text {and}\quad \mathcal {C}, X \vDash \lnot p \text { iff } f[\mathcal {C}] \vDash f(X)(d_p) \preceq \textbf{f}_p. \end{aligned}$$

#### Proof

We begin by noting that for a partial valuation *Y* of *F* we have

6.2 $$\begin{aligned} {{\,\textrm{dom}\,}}(f(Y))&= \{d_q \in D \mid q \in {{\,\textrm{dom}\,}}(Y)\}, \nonumber \\ {{\,\textrm{supp}\,}}(f(Y))&= \{d_q \in {{\,\textrm{dom}\,}}(f(Y)) \mid f(Y)(d_q) \text { is not the least element of }d_q\} \nonumber \\&= \{d_q \in D \mid q \in {{\,\textrm{dom}\,}}(Y) \text { and } f(Y)(d_q) \not = \textbf{f}_q\} \nonumber \\&= \{d_q \in D \mid Y \vDash q\}, \end{aligned}$$

6.3 $$\begin{aligned} {{\,\textrm{opp}\,}}(f(Y))&= \{d_q \in D \mid Y \vDash \lnot q\}. \end{aligned}$$

We proceed by induction on the position of *p* in the hierarchy $\textsf{H}$. In the base case, *p* is base-level and therefore so is $d_p$, we thus have

$$\begin{aligned}&\mathcal {C}, X \vDash p \\&\text {iff }X \vDash p&(\text {Lemmas}~22 \text { and } 23)\\&\text {iff }f(X)(d_p) = \textbf{t}_p&(\text {def}.\text { of } f(X))\\&\text {iff }\textbf{t}_p \preceq f(X)(d_p)&(\text {def}.\ \text { of } \preceq \text { on } d_p) \\&\text {iff }\mathcal {C}\vDash \textbf{t}_p \preceq f(X)(d_p).&(\text {Lemmas}~41 \text { and } 42) \end{aligned}$$

The other equivalence follows the same pattern.

In the induction case, when p∈A, we know that $\mathcal {C}, X \vDash q \text { iff } f[\mathcal {C}] \vDash \textbf{t}_q \preceq f(X)(d_q)$ and $\mathcal {C}, X \vDash \lnot q \text { iff } f[\mathcal {C}] \vDash f(X)(d_q) \preceq \textbf{f}_q$ for every $q \in \textsf{Pro}(p) \cup \textsf{Con}(p)$; now,

$$\begin{aligned}&f[\mathcal {C}] \vDash \textbf{t}_p \preceq f(X)(d_p) \\&\text {iff }\bullet \textbf{t}_p \preceq f(X)(d_p), \text { or} \\&\bullet \text { there is }Y \in f[\mathcal {C}] \text { such that }\textbf{t}_p \preceq Y(d_p) \text { and} \\&\quad \quad \,\,\,\,\text { -- for all }d_q \in \textsf{Pro}'(d_p) \cap {{\,\textrm{supp}\,}}(Y)\!\!: f[\mathcal {C}] \vDash Y(d_q) \preceq f(X)(d_q), \\&\quad \quad \,\,\,\,\text { -- for all }d_q \in \textsf{Con}'(d_p) \cap {{\,\textrm{opp}\,}}(Y)\!\!: f[\mathcal {C}] \vDash f(X)(d_q) \preceq Y(d_q) \\&\text {iff }\bullet \textbf{t}_p = f(X)(d_p), \text { or} \\&\bullet \text { there is } Y \in \mathcal {C}\text { such that } \textbf{t}_p \preceq f(Y)(d_p) \text { and} \\&\quad \quad \,\,\,\,\text { -- for all }d_q \in \textsf{Pro}'(d_p) \cap {{\,\textrm{supp}\,}}(f(Y))\!\!: f[\mathcal {C}] \vDash f(Y)(d_q) \preceq f(X)(d_q), \\&\quad \quad \,\,\,\,\text { -- for all }d_q \in \textsf{Con}'(d_p) \cap {{\,\textrm{opp}\,}}(f(Y))\!\!: f[\mathcal {C}] \vDash f(X)(d_q) \preceq f(Y)(d_q). \end{aligned}$$

We now apply Equations (6.2) and (6.3), and fill in $f(Y)(d_q)$, to continue with:

$$\begin{aligned}&\text {iff }\bullet X \vDash p, \text { or} \\&\quad \bullet \text {there is }Y \in \mathcal {C}\text { such that } Y \vDash p \text { and} \\&\quad \qquad \text {-- for all } d_q \in D \text { such that }q \in \textsf{Pro}(p) \text { and }Y \vDash q\!\!: f[\mathcal {C}] \vDash \textbf{t}_q \preceq f(X)(d_q), \\&\quad \qquad \text {-- for all }d_q \in D \text { such that }q \in \textsf{Con}(p) \text { and } Y \vDash \lnot q\!\!: f[\mathcal {C}] \vDash f(X)(d_q) \preceq \textbf{f}_q. \end{aligned}$$

This means we can apply the induction hypothesis to obtain:

$$\begin{aligned}&\text {iff }\bullet X \vDash p, \text { or} \\&\quad \bullet \text {there is }Y \in \mathcal {C}\text { such that } Y \vDash p \text { and} \\&\qquad \quad \text {-- for all } q \in \textsf{Pro}(p)\!\!:\text { if }Y \vDash q \text { then }\mathcal {C}, X \vDash q, \\&\qquad \quad \text {-- for all } q \in \textsf{Con}(p)\!\!:\text { if }Y \vDash \lnot q \text { then }C, X \vDash \lnot q \\&\text {iff }\mathcal {C}, X \vDash p. \end{aligned}$$

The other equivalence can be derived in the same manner, which completes the induction case and thus the proof. □

### Relation to the DRM

We now show that the dhrm is a conservative extension of the drm, in the sense that when it is restricted to flat dimensions hierarchies, with only one ‘outcome’ issue, it reduces to the drm. To show this, we will construct a translation *f* that maps instances of the drm to instances of the dhrm, and prove that this translation respects Definitions 32 and 39 of constraint. As in Sect. 6.5, we use the notion of lower bounding to a maximal element (or upper bounding to a minimal element) to play the role of forcing specific outcomes. In other words, a decision for an outcome dimension $d_\pi$ is lower bounded to $\textbf{t}$ in the translated dhrm instance if and only if the decision was forced for π in the original drm instance.

#### Definition 49

A dimension hierarchy is *flat* if its set of abstract dimensions is a singleton, and *issue binary* if its issues are binary dimensions in the sense of Definition 36.

Let *D* be a set of dimensions. We define an ‘outcome dimension’ $d_\pi = \{\textbf{t}, \textbf{f}\}$, ordered by the reflexive closure of $\textbf{f}\prec \textbf{t}$, and relations $\textsf{Pro}$ and $\textsf{Con}$ on $D \cup \{d_\pi \}$ by $\textsf{Pro}= D \times \{d_\pi \}$ and $\textsf{Con}= \emptyset$, so that we have a flat, issue binary dimension hierarchy $f(D) = (D \cup \{d_\pi \}, \textsf{Pro}, \textsf{Con})$ (the choice of link polarity is arbitrary here, cf. Remark 44). Next, we extend *f* to operate on fact situations, cases, and case bases. Given a fact situation *X* for the drm (with respect to the set of dimensions *D*) we translate it to a fact situation *f*(*X*) for the dhrm (with respect to the dimension hierarchy *f*(*D*)) by defining f(X)(d)=X(d) if $d \in {{\,\textrm{dom}\,}}(X)$, and otherwise leaving *f*(*X*)(*d*) undefined. For a case (*X*, *s*) we extend *f*(*X*) to a choice function *f*(*X*, *s*) on $D \cup \{d_\pi \}$ by:

$$\begin{aligned} f(X, s)(d) = {\left\{ \begin{array}{ll} X(d) & \text {if }d \in {{\,\textrm{dom}\,}}(X), \\ \textbf{t}& \text {if }d = d_\pi \text { and }s = \pi , \\ \textbf{f}& \text {if }d = d_\pi \text { and }s = \delta . \end{array}\right. } \end{aligned}$$

As before, we write $f[\mathcal {C}] = \{f(X, s) \mid (X, s) \in \mathcal {C}\}$.

The translation *f* preserves and reflects constraint, in the following sense.

#### Theorem 50

Given a case base $\mathcal {C}$ for a factor partition $F = {{\,\textrm{Pro}\,}}\cup {{\,\textrm{Con}\,}}$ and a focus fact situation *X* we have

$$\begin{aligned} \mathcal {C}, X \vDash \pi \text { iff } f[\mathcal {C}] \vDash \textbf{t}\preceq f(X)(d_\pi ) \quad \text {and}\quad \mathcal {C}, X \vDash \delta \text { iff } f[\mathcal {C}] \vDash f(X)(d_\pi ) \preceq \textbf{f}\end{aligned}$$

#### Proof

We begin by noting that for a case $(Y, \pi ) \in \mathcal {C}$ we have

$$\begin{aligned} {{\,\textrm{dom}\,}}(f(Y, \pi ))&= {{\,\textrm{dom}\,}}(Y) \cup \{d_\pi \}, \\ \textsf{Pro}(d_\pi )&= D, \\ \textsf{Con}(d_\pi )&= \emptyset , \text { and so} \\ \textsf{Pro}(d_\pi ) \cap {{\,\textrm{supp}\,}}(f(Y, \pi ))&= {{\,\textrm{supp}\,}}(Y), \\ \textsf{Con}(d_\pi ) \cap {{\,\textrm{opp}\,}}(f(Y, \pi ))&= \emptyset . \end{aligned}$$

We now derive the equivalence $\mathcal {C}, X \vDash \pi$ iff $f[\mathcal {C}] \vDash \textbf{t}\preceq f(X)(d_\pi )$:

$$\begin{aligned}&f[\mathcal {C}] \vDash \textbf{t}\preceq f(X)(d_\pi ) \\&\text {iff } \bullet \textbf{t}\preceq f(X)(d_\pi ), \text { or } \\&\bullet d_\pi \in A \text { and there is } Y \in f[\mathcal {C}] \text { such that } \textbf{t}\preceq Y(d_\pi ) \text { and} \\&\quad \text {-- for all } d \in \textsf{Pro}(d_\pi ) \cap {{\,\textrm{supp}\,}}(Y)\!\!: f[\mathcal {C}] \vDash Y(d) \preceq f(X)(d), \\&\quad \text {-- for all }d \in \textsf{Con}(d_\pi ) \cap {{\,\textrm{opp}\,}}(Y)\!\!: f[\mathcal {C}] \vDash f(X)(d) \preceq Y(d) \\&\text {iff there is }(Y, s) \in \mathcal {C}\text { such that }\textbf{t}\preceq f(Y,s)(d_\pi )\text { and} \\&\text {-- for all }d \in \textsf{Pro}(d_\pi ) \cap {{\,\textrm{supp}\,}}(f(Y, s))\!\!: f[\mathcal {C}] \vDash f(Y, s)(d) \preceq f(X)(d), \\&\text {-- for all }d \in \textsf{Con}(d_\pi ) \cap {{\,\textrm{opp}\,}}(f(Y, s))\!\!: f[\mathcal {C}] \vDash f(X)(d) \preceq f(Y, s)(d) \\&\text {iff there is }(Y, \pi ) \in \mathcal {C}\text { such that} \\&\text {-- for all }d \in {{\,\textrm{supp}\,}}(Y)\!\!: f[\mathcal {C}] \vDash f(Y, s)(d) \preceq f(X)(d) \\&\text {iff there is }(Y, \pi ) \in \mathcal {C}\text { such that for all }d \in {{\,\textrm{supp}\,}}(Y)\!\!: Y(d) \preceq X(d) \\&\text {iff }\mathcal {C}, X \vDash \pi . \end{aligned}$$

The derivation of $\mathcal {C}, X \vDash \delta$ iff $f[\mathcal {C}] \vDash f(X)(d_\pi ) \preceq \textbf{f}$ is very similar, so we omit it.

## Conclusion

In this work we have extended different versions of Horty’s (2011; 2019) models of a fortiori reasoning to incorporate both hierarchical and dimensional information. This has resulted in four different models, of which we analyzed the formal properties. Most notably, we found each to constitute monotonic forms of reasoning (with respect to both the addition of new cases and of new facts), and showed they are conservative extensions of one another. Ultimately, we hope to be able to apply these models to the task of making data-driven machine learning systems more interpretable.

Lastly, we mention some ideas for future work. Firstly, we aspire to operationalize the dhrm, in that we want to develop code for computing with it. Recently, van Woerkom et al. (2024) have used a logical formulation of the drm to implement it in the SMT solver Z3 (de Moura and Bjørner 2008), and used this to evaluate its fit to (real world) data. We think the dhrm admits a very similar logical specification, meaning it, too, can be implemented using the Z3 solver. We intend to make such an implementation, and subsequently evaluate it on data about bail decisions, which fits the running example we used in this work. Fortunately, such data was recently made available by Williams and Kolter (2021), which contains the same dimensions appearing in our running example, and so lends itself well to such an application.

Secondly, there is also more work to be done on the theoretical side. There is a well-known relation between Horty’s result and reason models, which is that their induced constraints coincide when the entire set of pro factors is taken as the reason for the decision of a case (Horty, 2011, Observation 6). In other words, the result model can be obtained from the reason model by restricting the reasons to be the entire set of pro factors. The same can be done at the hierarchical level, and so in this way the hierarchical reason model of Canavotto and Horty (2023b) yields an alternative definition to the hierarchical result model which we proposed in Sect. 4. It would be interesting to investigate precisely what the relation is between these two approaches.

## Footnotes

1 For example, in recent years there has been much discussion surrounding allegations put forth in Angwin et al. (2016) that the compas system, widely used in the United States for automatic recidivism risk assessment, was making racially biased decisions.

2 Factors that can have more than two possible values, such as the “amount of money expended” in this example, are better known as *dimensions* in the literature.

3 This is an updated version of the definition used by van Woerkom et al. (2023b, 2023a), which contains the implication “if X⊨p then Y⊨p” for the con factors of the precedent case.

4 This notation is shorthand for ‘support’ and ‘opposition’. Function support is a set-theoretic notion, where the support ${{\,\textrm{supp}\,}}(f)$ of a function $f: X \rightarrow \mathbb {R}$ from a set *X* to the real numbers $\mathbb {R}$ is defined as the subset of *X* that is not mapped to 0 by *f*; i.e. ${{\,\textrm{supp}\,}}(f) = \{x \in X \mid f(x) \not = 0\}$.
